# Identifying and validating subtypes within major psychiatric disorders based on frontal–posterior functional imbalance via deep learning

**DOI:** 10.1038/s41380-020-00892-3

**Published:** 2020-10-01

**Authors:** Miao Chang, Fay Y. Womer, Xiaohong Gong, Xi Chen, Lili Tang, Ruiqi Feng, Shuai Dong, Jia Duan, Yifan Chen, Ran Zhang, Yang Wang, Sihua Ren, Yi Wang, Jujiao Kang, Zhiyang Yin, Yange Wei, Shengnan Wei, Xiaowei Jiang, Ke Xu, Bo Cao, Yanbo Zhang, Weixiong Zhang, Yanqing Tang, Xizhe Zhang, Fei Wang

**Affiliations:** 1 Department of Radiology, The First Affiliated Hospital of China Medical University, Shenyang, China; 2 Department of Psychiatry, Washington University School of Medicine, St. Louis, MO USA; 3 State Key Laboratory of Genetic Engineering and Human Phenome Institute, School of Life Sciences, Fudan University, Shanghai, China; 4 School of Computer Science and Engineering, Northeastern University, Shenyang, Liaoning China; 5 Department of Psychiatry, The First Affiliated Hospital of China Medical University, Shenyang, China; 6 Shanghai Center for Mathematical Science, Fudan University, Shanghai, China; 7 Department of Psychiatry, University of Alberta, Edmonton, AB Canada; 8 Department of Psychiatry, College of Medicine University of Saskatchewan Ellis Hall, Royal University Hospital, Saskatoon, SK Canada; 9 Department of Computer Science and Engineering, Washington University, St. Louis, MO USA; 10 Department of Genetics, Washington University School of Medicine, St. Louis, MO USA; 11 School of Biomedical Engineering and Informatics, Nanjing Medical University, Nanjing, Jiangsu China; 12 Nanjing Brain Hospital, Nanjing Medical University, Nanjing, Jiangsu China

**Keywords:** Psychology, Prognostic markers, Neuroscience

## Abstract

Converging evidence increasingly implicates shared etiologic and pathophysiological characteristics among major psychiatric disorders (MPDs), such as schizophrenia (SZ), bipolar disorder (BD), and major depressive disorder (MDD). Examining the neurobiology of the psychotic-affective spectrum may greatly advance biological determination of psychiatric diagnosis, which is critical for the development of more effective treatments. In this study, ensemble clustering was developed to identify subtypes within a trans-diagnostic sample of MPDs. Whole brain amplitude of low-frequency fluctuations (ALFF) was used to extract the low-dimensional features for clustering in a total of 944 participants: 581 psychiatric patients (193 with SZ, 171 with BD, and 217 with MDD) and 363 healthy controls (HC). We identified two subtypes with differentiating patterns of functional imbalance between frontal and posterior brain regions, as compared to HC: (1) Archetypal MPDs (60% of MPDs) had increased frontal and decreased posterior ALFF, and decreased cortical thickness and white matter integrity in multiple brain regions that were associated with increased polygenic risk scores and enriched risk gene expression in brain tissues; (2) Atypical MPDs (40% of MPDs) had decreased frontal and increased posterior ALFF with no associated alterations in validity measures. Medicated Archetypal MPDs had lower symptom severity than their unmedicated counterparts; whereas medicated and unmedicated Atypical MPDs had no differences in symptom scores. Our findings suggest that frontal versus posterior functional imbalance as measured by ALFF is a novel putative trans-diagnostic biomarker differentiating subtypes of MPDs that could have implications for precision medicine.

## Introduction

Definitive biomarkers have remained elusive in psychiatry, while other fields of medicine have amassed an armory of biomarkers for diagnosis and treatment. This is not entirely surprising as studies have primarily utilized nosology that differentiates neuropsychiatric disorders based on clinical phenomenology in the absence of any biological determinant, albeit the *Diagnostic and Statistical Manual of Mental Disorders* (DSM) has revolutionized the field and advanced it to its current state. Long conceptualized as distinct diagnostic categories, major psychiatric disorders (MPDs), consisting of schizophrenia (SZ), bipolar disorder (BD), and major depressive disorder (MDD), share substantial core features as implicated by converging lines of evidence from genetic, molecular, histological, and neuroimaging studies [1–6]. Thus, there appears to be a greater continuum between psychotic and affective disorders than previously thought. Consequently, understanding the core changes in MPDs is critical for mapping the principal neural pathways resulting in psychopathology and the crossroads at which divergent paths lead to varying clinical phenomenology within and across diagnoses.

Several studies have adopted alternative approaches to identifying brain-based biomarkers that transcend traditional diagnostic boundaries [7, 8]. Recently, Clementz et al. conducted a k-means clustering analysis of cognitive and electrophysiological measures using trans-diagnostic data generated from the Bipolar-Schizophrenia Network for Intermediate Phenotype consortium [7]. They identified three “biotypes” that were largely orthogonal to the DSM-IV diagnoses and significantly different with respect to external validating measures such as brain structure and function [9, 10]. Their approach has been touted as an important step toward a more neurobiologically based understanding of psychosis [11]. Subsequently, pioneering work by Drysdale et al. identified four biotypes in depression using canonical correlation analysis of the Hamilton Depression Rating Scale (HAMD) to characterize connectivity features [8]. Their work presented yet another strategy for refining classification within clinically heterogeneous diagnoses, as well as identifying individuals who may be more responsive to transcranial magnetic stimulation.

Neuroimaging has offered a wealth of potential biomarkers for neuropsychiatric disorders. Abnormal brain function has been proven to be useful in the assessment of pain [12] and shows great promise for application to neuropsychiatric illnesses. Resting-state functional magnetic resonance imaging is well-established and has been widely performed for noninvasive exploration of the brain’s intrinsic functional architecture using measurements of spontaneous low-frequency fluctuations (LFFs) in the blood oxygenation level-dependent (BOLD) signal [13, 14]. Although their underlying mechanism is not exactly clear, LFFs appear to arise from neurovascular activity [15] and have been associated with glutamatergic/GABAergic synaptic currents and glial activity [16, 17]. Furthermore, the amplitude of BOLD signal fluctuations is proportional to regional cerebral blood flow, which is an established marker of brain metabolic activity [18]. The amplitude of low-frequency fluctuations (ALFF; generally in the range of 0.01–0.08 Hz) appears to be an efficient index of local spontaneous neuronal activity at rest [19]. Regional variability in ALFF reflects spontaneous fluctuations in a given voxel independent of its neighboring, regional, or network connectivity. Moreover, ALFF exhibits moderate to substantial test-retest reliability [20] ensuring a high upper bound for its validity as a regional functional measure to detect individual differences [21]. Prior studies, including a multi-center study and our previous work, have shown significant alterations in ALFF across MPDs compared to healthy controls (HC), most prominently in frontal, subcortical, and temporal regions, as well as in visual regions (precuneus and cuneus); however, inconsistencies have been reported [6, 22, 23].

In this study, we present a novel clustering method utilizing deep learning to identify subtypes across the psychotic-affective disorder spectrum in a trans-diagnostic sample of MPDs. We used a deep stacked *AutoEncoder* to extract low-dimensional features of ALFF followed by an ensemble clustering method to identify ALFF-based subtypes that were maximally dissimilar from each other in MPDs. We then validated the resulting subtypes using cortical thickness, white matter integrity as measured by fractional anisotropy (FA), polygenic risk scores (PRS), and risk gene expression tissue profile. We also examined the effects of medication status on symptom severity to elucidate possible pharmacologic effects within each of the subtypes.

## Methods

### Samples and measures

The study included a total of 944 participants consisting of 581 patients with MPDs (193 with SZ, 171 with BD, 217 with MDD) and 363 HC, who were recruited and scanned at a single site with identical inclusion and exclusion criteria. MPD participants were recruited from the inpatient and outpatient services at Shenyang Mental Health Center and Department of Psychiatry, The First Affiliated Hospital of China Medical University, Shenyang, China. HC participants were recruited from the local community by advertisement. Behavioral symptoms were assessed using the HAMD and Brief Psychiatric Rating Scale (BPRS). Cognitive function was assessed using the Wisconsin Card Sorting Test (WCST). Demographic and clinical characteristics are detailed in Supplementary Tables 1 and 2. Whole blood samples (243 patients and 193 HC) were collected. All participants provided written informed consent after receiving a detailed description of the study. The study was approved by the Institutional Review Board of China Medical University.

Functional MRI, structural MRI, and diffusion tensor imaging (DTI) were acquired in a GE Signa HD 3.0T scanner with a standard 8-channel head coil at the First Affiliated Hospital of China Medical University, Shenyang, China. Functional images were collected with a gradient echo planar imaging (EPI) sequence for ALFF measures. Three-dimensional, high-resolution, T1-weighted images were collected using a 3-D fast spoiled gradient echo sequence to measure cortical thickness. DTI used a single-short spin-EPI sequence to measure FA for assessing white matter integrity. For all scanning sequence parameters and image preprocessing, please see “Methods” in the Supplementary information.

Genotyping, imputation and calculation of PRS, and risk gene expression were also performed. Details about how they were performed can be found in “Methods” in the Supplementary information.

### Ensemble clustering method based on deep learning

**Fig. 1:**
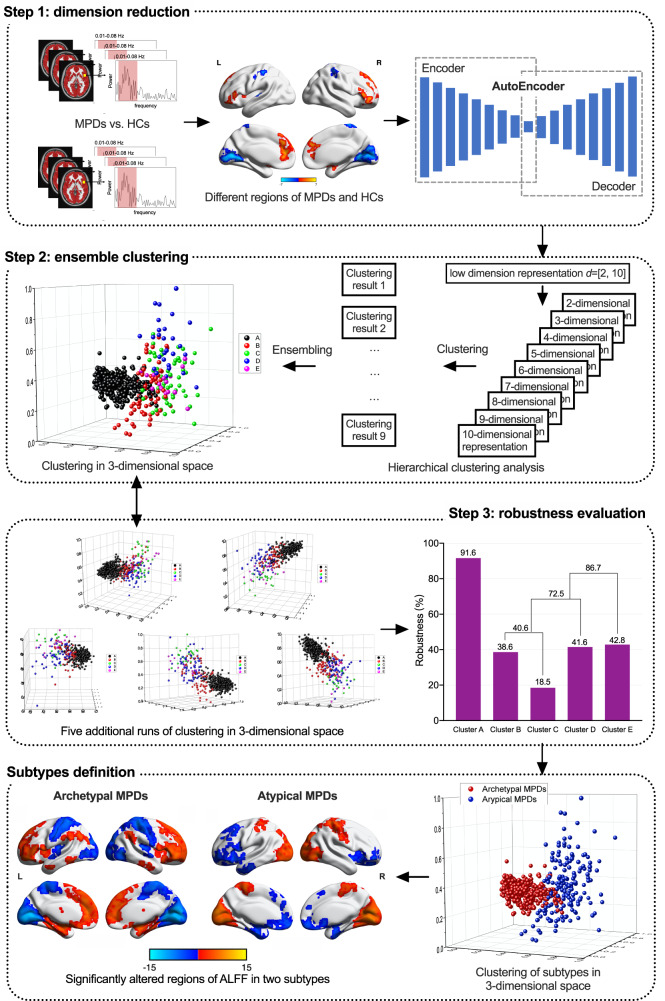
Schematic of using deep learning-based hierarchical clustering to define clusters of MPDs. Step one: identification of significant functional alterations in MPDs and using *AutoEncoder* to reduce the dimension of the identified alterations to *d* ∈ [2,10]. Step two: for each of the nine low-dimensional data from step one, we obtained nine different class labels based on clustering analyses, and five clusters (cluster A, B, C, D, and E) were identified. Step three: we performed the clusters merging process according to six runs of clustering and obtained two final subtypes. Furthermore, the subtypes varied in patterns of amplitude of low-frequency fluctuation alterations as compared to HC (voxel *p* < 0.001 with Gaussian random field correction for cluster *p* < 0.05). MPD major psychiatric disorder; HC healthy control; L left; R right; d dimension.

Clustering algorithms group data points (i.e., participants) based on their similarity in dimensional space. For high-dimensional data, such as whole brain ALFF, the number of dimensions must be reduced to avoid the “curse of dimensionality” [24]. Consequently, clustering results are dependent on the dimensional representation selected for analyses; however, there is no established standard for selecting an appropriate dimensional representation. Thus, our algorithm was designed to perform clustering analyses for multiple dimensional representations and then use consensus group assignment followed by a robustness optimization protocol to achieve the most reliable and stable subtype assignment. Subtypes were identified in *n* = 581 patients with MPDs using a novel ensemble clustering method based on deep stacked AutoEncoder in the following steps (Fig. 1).

#### Step one: dimension reduction

To identify principal ALFF alterations, we extracted voxels with significantly different ALFF between MPDs and HC using a general linear model (GLM). For GLM analyses, gender (male/female) and group (MPDs/HC) were included as discrete factors and age as a continuous factor, and the effect of group on ALFF was the primary interest of the analysis. The significance level was set at voxel *p* < 0.001 with the Gaussian random field (GRF) correction for cluster *p* < 0.05. A total of 2175 voxels were identified as significantly different in ALFF between MPDs and HC from the whole brain of 42,185 voxels. We then used AutoEncoder [25], a deep artificial neural network, to further reduce the dimensions of the input data to *d* ∈ [2, 10]. AutoEncoder included an encoder and a symmetric decoder. The encoder compressed the 2175 voxels obtained as described above into a low-dimensional representation consisting of nine layers with sizes 2175-2048-1024-512-256-128-64-32-*d* and the symmetrical reconstruction by the decoder as the output. Mean square error was used as the loss function to minimize the differences between the input of 2175 voxels and the reconstructed voxels at the output layer. Compared to the conventional dimensionality reduction methods such as principal component analysis, AutoEncoder is capable of learning intrinsic, nonlinear relationships in the input data and therefore better suited for high-dimensional nonlinear data [25].

#### Step two: ensemble clustering

A common problem of clustering high-dimensional data is that inappropriate low-dimensional representations of data will lead to unreliable clustering results. To avoid this problem, we designed a new ensemble method to integrate hierarchical clustering results from multiple d-dimensional representations (*d* ∈ [2, 10]) (Supplementary Fig. 1) which obtained from the autoencoder. The Euclidean distance was used to compute the distance between participants and the complete linkage method was used to compute the distance between clusters. For each d-dimensional representation (*d* ∈ [2, 10]), we obtained a set of clustering results for all participants. Therefore, each participant was subsequently assigned nine class labels, one for each of the clustering result based on nine d-dimensional representations. A consensus was determined by the majority of the nine class labels and served as the cluster assignment for each participant. Therefore, the ensembled result can better reflect the inherent clustering characteristics of the data, because it integrates the results from multiple low-dimensional representations of participants.

#### Step three: optimization of clustering robustness

While the ensemble clustering method was effective, it was relatively sensitive to the low-dimensional representations obtained from the autoencoder. To improve the robustness of the clustering results, we merged some clusters based on multiple runs of the clustering method. To this end, we first introduced a new index to quantify the robustness of a cluster. The robustness index *R*_*i*_ of cluster *i* was calculated as $R_i = \frac{{| { \cap _jC_i^j} |}}{{| { \cup _jC_i^j} |}}$, where $C_i^j$ is the *j*-th run of the clustering method on cluster *i*. A larger robustness index *R*_*i*_ means a more stable cluster *i*. We then adopted an iterative, hierarchical scheme to merge clusters with low robustness. Specifically, we iteratively combined the two clusters with the lowest robustness indices until all clusters were robust enough, i.e., their robustness indices were greater than a threshold *δ*. In our analysis, *δ* was set to 0.8 based on our experiment on the brain image data.

### Subtype-related validation across multi-level biological data

#### Comparison of ALFF alterations with HC

Group comparisons of ALFF values were performed by DPABI [26]. For each voxel, GLM was performed to examine the difference in ALFF between each subtype and HC. For GLM analyses, gender and group were included as discrete factors and age as a continuous factor, and the effect of group on ALFF was of primary interest. Statistical significance was determined by combining individual voxel *p* (uncorrected) < 0.001 with GRF correction for cluster-level inference of *p* < 0.05.

#### Cortical thickness and white matter integrity

Group comparisons of cortical thickness were performed vertex-wise on the cortical surface by *Freesurfer* (MRI_glmfit), and FA values were calculated in SPM8 (http://www.fil.ion.ucl.ac.uk/spm). For each vertex or voxel, GLM was used to examine differences in cortical thickness and FA between each subtype and HC. GLM design and statistical significance were the same as those in ALFF analyses.

#### Genetic loading analysis

Association of PRS (PRS-SZBD and PRS-MDD) with each subtype was performed with logistic regression, and Nagelkerke’s pseudo-*R*^2^ was calculated to measure the proportion of variance explained. We estimated and analyzed high-resolution PRS at 105 different levels of *P*_*T*_ (ranging from 0 to 0.5 with increments of 0.005 plus 10^−6^, 10^−5^, 10^−4^, 0.001, and 1). To correct for multiple comparison, a significance threshold of *p* = 0.004 was adopted as suggested by Euesden et al. [27].

#### Clinical and cognitive measures

Two-sample *t*-tests were used to examine differences in HAMD and BPRS total and factor scores and WCST scores between subtypes. Statistical significance was set at *p* < 0.05 with FDR correction for multiple comparisons. HAMD and BPRS factor scores were identified from exploratory factor analysis using the principal component factor method in MPDs (*n* = 581) (“Methods” in Supplementary information, Supplementary Tables 3 and 4). Subsequently, the resulting HAMD and BPRS factors were used in a group analysis where we performed two-sample *t*-tests (*p* < 0.05 with FDR correction) to examine the effects of medication status for each subtype.

### Clinical diagnosis-related validation across multi-level biological data

We also performed analogous analyses on ALFF, cortical thickness, white matter integrity, PRS, risk gene expression, and effects of medication status based on clinical diagnosis.

## Results

### Identified subtypes **and relation to clinical diagnoses**

The novel ensemble clustering method identified two subtypes in the MPDs sample (*n* = 581), Archetypal MPDs (cluster A, 60% of the MPDs sample) and Atypical MPDs (Fig. 1 and Supplementary Table 5). The distribution of clinical diagnosis (SZ, BD, and MDD) varied between Archetypal and Atypical MPDs. A greater proportion of SZ appeared in Archetypal MPDs (40%) than Atypical MPDs (16%). BD and MDD represented 27% and 33%, respectively, in Archetypal MPDs and 35% and 49%, respectively, in Atypical MPDs (Supplementary Fig. 2a). From the perspective of clinical diagnoses, there were more SZ belong to Archetypal MPDs (86%). While the proportion of BD and MDD subtyped as Archetypal MPDs were relatively smaller (65 and 61%, respectively), BD and MDD comprised much larger portions of Atypical MPDs than SZ. 86% of SZ were subtyped asArchetypal Mods (Supplementary Fig. 2b).

### Subtype-related characteristics

#### Subtype-related ALFF alterations

In Archetypal MPDs (*n* = 411), ALFF was significantly increased in frontal areas (prefrontal cortex, limbic, paralimbic, and striatum) and significantly decreased in posterior areas (primary sensory and motor cortices and unimodal association cortices), compared to HC (*n* = 363) (Cohen’s *d* = 0.64, *p* < 0.001) (Subtypes definition in Fig. 1). The converse was observed in Atypical MPDs (*n* = 170): ALFF was significantly decreased in frontal areas (prefrontal cortex, limbic, paralimbic, and striatum), and was significantly increased in posterior areas (primary sensory and motor cortices and unimodal association cortices), compared to HC (*n* = 363) (Cohen’s *d* = 0.43, *p* < 0.001) (Subtypes definition in Fig. 1).

#### Subtype-related cortical thickness and white matter integrity

**Fig. 2:**
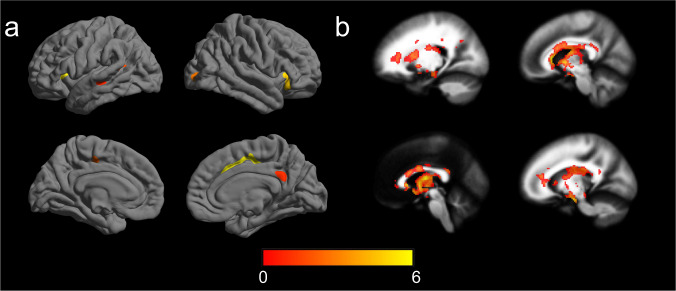
Significant differences in (a) cortical thickness and (b) white matter integrity between Archetypal MPDs and healthy controls. Significance level was set to voxel *p* < 0.001 with Gaussian random field correction for cluster *p* < 0.05. The color bar represents *t* value. MPD major psychiatric disorder.

In Archetypal MPDs, cortical thickness (*n* = 377) and FA values (*n* = 397) were significantly decreased in multiple brain regions compared to HC (*n* = 353 and 359) (cortical thickness: Cohen’ *d* = 0.28; *p* = 0.002. FA values: Cohen’ *d* = 0.52; *p* < 0.001) (Fig. 2). In Atypical MPDs, no significant differences in cortical thickness (*n* = 159) and FA values (*n* = 164) were observed compared to HC (*n* = 353 and 359).

#### Subtype-related polygenetic risk

**Fig. 3:**
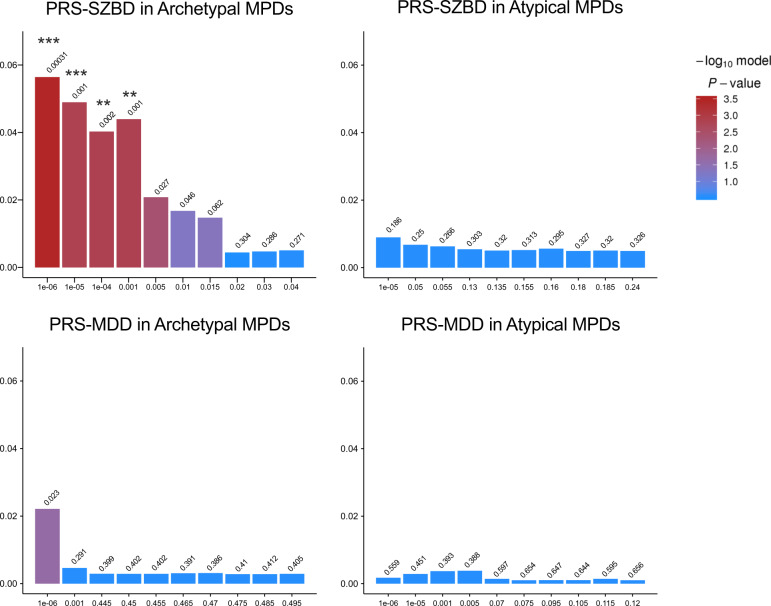
The variance (*y*-axis) of case-control status explained by the PRS-SZBD and PRS-MDD in Archetypal and Atypical MPDs. *x*-axis represents *p* value threshold, *y*-axis represents PRS model fit: *R*^2^ (Nagelkerke’s). The bars represent ten best-fit PRS scores calculated at different *p* value threshold. ***p < 0.001; **p < 0.01. PRS-SZBD, polygenetic risk score of schizophrenia and bipolar disorder, PRS-MDD, polygenetic risk score of major depressive disorder. MPD major psychiatric disorder.

Four PRS-SZBD scores at *P*_*T*_ of 10^−6^ (*N*_SNPs_ = 300), 10^−5^ (*N*_SNPs_ = 565), 10^−4^ (*N*_SNPs_ = 1203), and 0.001 (*N*_SNPs_ = 2978) showed significant differences between Archetypal MPDs (*n* = 143) and HC (*n* = 192), explaining 5.6%, 4.9%, 4.3%, and 4.0%, respectively, of the variation in Archetypal MPDs. The scores remained significant after multiple comparison correction. Compared to HC, no significant difference was observed in PRS-SZBD in Atypical MPDs (*n* = 100) or PRS-MDD in either subtypes. The ten best-fit PRS scores for each subtype are presented in Fig. 3.

#### Subtype-related gene expression

**Fig. 4:**
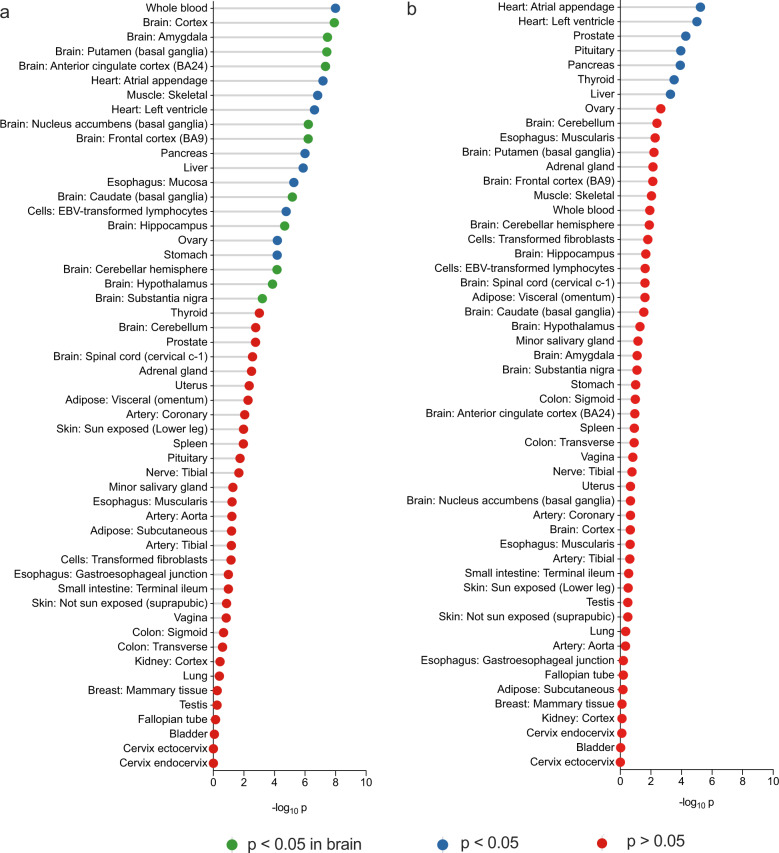
Differentially expressed risk genes across 53 tissues in (a) Archetypal and (b) Atypical MPDs. MPD major psychiatric disorder.

Combining GWAS data and frontal cortex eQTL, we identified 173 genes significantly associated with Archetypal MPDs (*n* = 143) and 138 genes with Atypical MPD (*n* = 100) (Supplementary Excel 1). These genes were then used as input to an expression enrichment analysis on the web-based tool, FUMA. The two sets of genes showed differential expression profiles across 53 human tissues from GTEx [28]. Archetypal MPDs-associated genes were significantly expressed in 21 tissues; about half (11 tissues) represent brain tissues (Fig. 4a). The genes associated with Atypical MPDs were predominantly expressed in nonbrain tissues including the heart, prostate, pituitary, pancreas, thyroid, and liver (Fig. 4b).

#### Clinical characteristics within subtypes

**Fig. 5:**
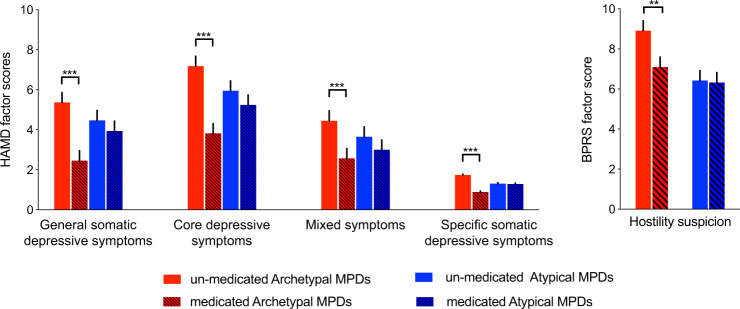
Significant differences in HAMD factors and BPRS factor scores between medicated and unmedicated patients in Archetypal and Atypical MPDs. The significance level was set to *p* < 0.05 with false discovery rate correction. Vertical black lines show the standard errors of the means. ***p < 0.001; **p < 0.01. HAMD Hamilton Depression Scale; BPRS Brief Psychiatric Rating Scale; MPD major psychiatric disorder.

Medicated Archetypal MPDs had significantly decreased HAMD and BPRS factor scores than their unmedicated counterpart (Fig. 5). Factors that differed significantly were general somatic depressive symptoms (*n* = 377; 95% CI, 2.24–3.58; Cohen’s *d* = 0.94; *p* < 0.001), core depressive symptoms (*n* = 377; 95% CI, 2.37–4.36; Cohen’s *d* = 0.70; *p* < 0.001), somatization (*n* = 377; 95% CI, 1.26–2.51; Cohen’s *d* = 0.66; *p* < 0.001), and mixed symptoms (retardation, agitation, psychiatric anxiety, and insight) (*n* = 377; 95% CI, 0.52–1.17; Cohen’s *d* = 0.59; *p* < 0.001) in the HAMD; and hostility and suspicion in the BPRS (*n* = 306; 95% CI, 0.52–3.13; Cohen’s *d* = 0.36; *p* = 0.007) (Fig. 5). No significant differences in HAMD and BPRS factor scores were observed between medicated and unmedicated Atypical MPDs (Fig. 5).

### Biological and clinical characterization based on clinical diagnosis

Multimodal biological characterization based on clinical diagnosis showed continuum alterations across SZ, BD, and MDD. The details please see Supplementary results and Supplementary Figs. 3–7.

## Discussion

In this study, we developed a novel clustering method utilizing deep learning to identify two major ALFF-based subtypes, Archetypal MPDs and Atypical MPDs, that differed in genetic, multimodal MRI, and clinical characteristics. Archetypal MPDs (60% of the MPDs sample) had significantly increased ALFF in frontal areas (prefrontal cortex, limbic, paralimbic, and striatum) and significantly decreased ALFF in posterior areas (primary sensory and motor cortices and unimodal association cortices), significantly higher genetic vulnerability with increased PRS-SZBD, enriched risk gene expression in brain regions including the frontal cortex, limbic system, basal ganglia, hypothalamus, cerebellum, and substantia nigra, and significantly decreased cortical thickness and white matter integrity in multiple brain regions, compared to HCs. Medicated Archetypal MPDs had significantly decreased HAMD and BPRS factor scores than unmedicated Archetypal MPDs, suggesting the effect of medication status on symptom severity in this subtype. In contrast, Atypical MPDs (40%) were defined by significantly decreased ALFF in frontal regions and significantly increased ALFF in the posterior brain without associated differences in PRS scores, cortical thickness, or white matter integrity compared to HC. Risk gene expression was prominent in nonbrain tissues, such as heart, liver, pancreas, and pituitary, which are regarded as somatic and endocrine-related tissues. No significant differences in HAMD and BPRS factor scores were observed between medicated and unmedicated Atypical MPDs, suggesting the lack of medication status effects on symptom severity in the subtype. Collectively, our findings implicated functional imbalance between the frontal and posterior regions as a core and differentiating feature across the psychotic-affective disorder continuum. Furthermore, the subtypes, Archetypal and Atypical MPDs, delineated by this feature were differentially associated with genetic vulnerability, risk gene expression, cortical thickness, and white matter integrity. Interestingly, Archetypal and Atypical MPDs were also distinct in the effects of medication status on symptom severity, suggesting possible differential pharmacologic effects in the two subtypes. Additionally, multimodal biological characterization based on clinical diagnosis showed continuum alterations across SZ, BD, and MDD. These findings further support that there is a greater neurobiological overlap than previously thought among these clinical diagnoses.

The observed ALFF pattern in Archetypal MPDs was consistent with our prior findings of increased frontal and decreased posterior ALFF as a shared feature across SZ, BD, and MDD [6, 29, 30]. Significantly increased ALFF appeared in frontal regions including the prefrontal cortex, limbic, paralimbic, and striatum and significantly decreased ALFF in the posterior primary cortices in MPDs [6]. Moreover, we also found that the ALFF ratio between these regions in slow-4 was negatively correlated with measures of negative and disorganized symptoms across SZ, BD, and MDD [6]. Altogether, these findings suggest impaired balance between regions conventionally known for emotional perception and processing and the visual cortices in MPDs.

Studies utilizing trans-diagnostic approaches are emerging [5, 6, 9, 10] as converging evidence indicates core features across MPDs and increasing focus on the brain and neuropsychiatric disorders from a systems perspective (i.e., National Institute of Mental Health Research Domain Criteria). Our findings in this study defined two subtypes across clinical diagnostic boundaries. Each clinical diagnosis, SZ, BD, or MDD, was represented in each subtype reported herein. We also performed multimodal biological characterization based on clinical diagnosis and found continuum alterations across SZ, BD, and MDD (for details regarding methods and results, see Supplementary information). Altogether, these findings further support that there is a greater neurobiological overlap than previously thought among the three clinical diagnoses. The mismatch between subtypes and clinical diagnoses may in part explain frequent inconsistent results among studies based on clinical diagnosis. The constraints of our current diagnostic system are apparent [7, 9, 10, 31]. Refining the current diagnostic system with relevant biological measures (e.g., frontal and posterior ALFF imbalance) would yield more biologically homogeneous groups, which are critically important for developing more effective and personalized treatment. Along these lines, we developed and compared two classification models using a 3D convolutional neural network that categorized participants based on (1) subtypes, Archetypal and Atypical MPDs and (2) clinical diagnoses, SZ, BD, and MDD. The accuracy and precision for the subtype-based model were significantly higher than the model based on clinical diagnoses, underscoring that clinical diagnoses share more similar features and are less distinguishable from each other in the classification models. For full details regarding methods and results, please see Supplementary information and Supplementary Fig. 8.

The differences between the two subtypes, Archetypal and Atypical MPDs, in association with PRS for BD and SZ, risk gene expression, cortical thickness, and white matter integrity are of significant interest. Compared to HC, Archetypal MPDs had increased PRS-SZBD, indicating a greater genetic vulnerability in this subtype. Archetypal MPDs had enriched risk gene expression in the brain (e.g., frontal cortex, limbic system, basal ganglia, hypothalamus, cerebellum, and substantia nigra), whereas Atypical MPD had risk gene expression more prominent in somatic and endocrine-related tissues such as the heart, liver, pancreas, and pituitary. Significant decreases in cortical thickness and white matter integrity were found broadly in Archetypal MPDs but not in Atypical MPDs. Findings in Archetypal MPDs are consistent with previous studies of MPDs [4, 5, 32–34]. Decreased neuronal and glial density, and genetic and neurotransmitter alterations have been found in multiple brain regions including the anterior cingulate cortex, dorsolateral prefrontal cortex, and nucleus accumbens across SZ, BD, and MDD [35]. Genetic imaging studies in SZ and BD individuals and their relatives suggest decreased gray matter volume [32, 36] and white matter integrity [33, 37] as potential heritable biomarkers. Altogether, our findings support the Archetypal MPDs as a genetic and neurodevelopmental subtype of neuropsychiatric disorders. Further studies are needed to determine the nature of Atypical MPDs (e.g., stress-induced or stress diathesis) and better understand the biological implications of this group.

Intriguingly, medicated Archetypal MPDs had significantly decreased symptom severity as measured by the HAMD and BPRS than their unmedicated counterpart but no significant differences in symptom severity were observed in medicated versus unmedicated Atypical MPDs, suggesting differential pharmacologic effects between the subtypes. This is further supported by the findings that the associated decreases in cortical thickness and white matter integrity in Archetypal MPDs could represent pathological processes that are responsive to medications [38, 39] or direct compensatory effects of medications [40, 41]. Moreover, we found enriched risk gene expression in the brain in Archetypal MPDs but not in Atypical MPDs. Wang et al. previously identified a genetic profile in SZ similar to our Archetypal MPDs that consisted of brain-expressed, high-risk genes enriched for targets of approved drugs [42]. Altogether, these findings raise questions as to whether conventional pharmacologic treatment may be more effective in Archetypal than Atypical MPDs. Further studies are needed to examine differences in treatment response between the two subtypes.

### Limitations

There are several limitations in this study. We used a single biomarker (ALFF) approach in our clustering method to identify subtypes within MPDs. There are likely other relevant biomarkers for clustering and subtyping, and multimodal data could capitalize on cross-information of the existing data [43]. Future studies are needed to identify other relevant biomarkers and determine how best to combine different measures of multimodal brain imaging features in clustering analyses for psychiatric disorders. As well as the potential use of the identified neuroimaging markers for individualized prediction of clinical or cognitive measures [44]. Further, while there appears to be a biological mechanism underlying ALFF, the exact nature of the ALFF alterations observed herein are not clear. They could relate to factors such as the number of prior depressive/manic/psychotic episodes. Unfortunately, we did not collect specific data about prior illness episodes and were not able to examine the relationship between ALFF alterations and prior depressive/manic/psychotic episodes. The ALFF alterations could also relate to other fMRI and biological measures aside from cortical thickness and white matter integrity. For greater biological validity and predictive utility, other biological or clinical measures should be included in future studies of the subtypes described here. Moreover, some studies have found that dynamic functional features are more conducive to information related to different mental activity than static features [45]. These cannot be measured using static parameters. Future work should consider applying dynamic functional features to investigate the abnormal activity. In addition, factors such as participant inclusion criteria, the size of our cluster-discovery data set, and the ordinal nature of our clinical measures could have restricted our ability to identify other subtypes.

## Conclusion

In summary, our findings implicated functional imbalance between frontal and posterior regions as a core and differentiating feature among MPDs. These findings could have significant contributions to the development of biologically informed diagnostic classifications and treatment guidelines across the psychotic-affective disorder continuum.

## Supplementary information

SupplementalMaterials

Supplemental Materials Excel 1-Sherlock gene list_MPDs

## References

[1] Cross-Disorder Group of the Psychiatric Genomics Consortium. (2013). Identification of risk loci with shared effects on five major psychiatric disorders: a genome-wide analysis. Lancet.

[2] Garcia-Rizo C, Kirkpatrick B, Fernandez-Egea E, Oliveira C, Bernardo M (2016). Abnormal glycemic homeostasis at the onset of serious mental illnesses: a common pathway. Psychoneuroendocrinology.

[3] Goldsmith DR, Rapaport MH, Miller BJ (2016). A meta-analysis of blood cytokine network alterations in psychiatric patients: comparisons between schizophrenia, bipolar disorder and depression. Mol Psychiatry.

[4] Goodkind M, Eickhoff SB, Oathes DJ, Jiang Y, Chang A, Jones-Hagata LB (2015). Identification of a common neurobiological substrate for mental illness. JAMA Psychiatry.

[5] Chang M, Womer FY, Edmiston EK, Bai C, Zhou Q, Jiang X (2018). Neurobiological commonalities and distinctions among three major psychiatric diagnostic categories: a structural MRI study. Schizophr Bull.

[6] Chang M, Edmiston EK, Womer FY, Zhou Q, Wei S, Jiang X (2019). Spontaneous low-frequency fluctuations in the neural system for emotional perception in major psychiatric disorders: amplitude similarities and differences across frequency bands. J Psychiatry Neurosci.

[7] Clementz BA, Sweeney JA, Hamm JP, Ivleva EI, Ethridge LE, Pearlson GD (2016). Identification of distinct psychosis biotypes using brain-based biomarkers. Am J Psychiatry.

[8] Drysdale AT, Grosenick L, Downar J, Dunlop K, Mansouri F, Meng Y (2017). Resting-state connectivity biomarkers define neurophysiological subtypes of depression. Nat Med.

[9] Ivleva EI, Clementz BA, Dutcher AM, Arnold SJM, Jeon-Slaughter H, Aslan S (2017). Brain structure biomarkers in the psychosis biotypes: findings from the bipolar-schizophrenia network for intermediate phenotypes. Biol Psychiatry.

[10] Meda SA, Clementz BA, Sweeney JA, Keshavan MS, Tamminga CA, Ivleva EI (2016). Examining functional resting-state connectivity in psychosis and its subgroups in the bipolar-schizophrenia network on intermediate phenotypes cohort. Biol Psychiatry Cognit Neurosci Neuroimaging.

[11] Barch DM (2017). Biotypes: promise and pitfalls. Biol Psychiatry.

[12] Wager TD, Atlas LY, Lindquist MA, Roy M, Woo CW, Kross E (2013). An fMRI-based neurologic signature of physical pain. N Engl J Med.

[13] Patriat R, Molloy EK, Meier TB, Kirk GR, Nair VA, Meyerand ME (2013). The effect of resting condition on resting-state fMRI reliability and consistency: a comparison between resting with eyes open, closed, and fixated. Neuroimage.

[14] Zuo XN, Di Martino A, Kelly C, Shehzad ZE, Gee DG, Klein DF (2010). The oscillating brain: complex and reliable. Neuroimage.

[15] Biswal B, Yetkin FZ, Haughton VM, Hyde JS (1995). Functional connectivity in the motor cortex of resting human brain using echo-planar MRI. Magn Reson Med.

[16] Krishnan GP, Gonzalez OC, Bazhenov M (2018). Origin of slow spontaneous resting-state neuronal fluctuations in brain networks. Proc Natl Acad Sci USA.

[17] Nugent AC, Martinez A, D'Alfonso A, Zarate CA, Theodore WH (2015). The relationship between glucose metabolism, resting-state fMRI BOLD signal, and GABAA-binding potential: a preliminary study in healthy subjects and those with temporal lobe epilepsy. J Cereb Blood Flow Metab.

[18] Noda A, Ohba H, Kakiuchi T, Futatsubashi M, Tsukada H, Nishimura S (2002). Age-related changes in cerebral blood flow and glucose metabolism in conscious rhesus monkeys. Brain Res.

[19] Zang YF, He Y, Zhu CZ, Cao QJ, Sui MQ, Liang M (2007). Altered baseline brain activity in children with ADHD revealed by resting-state functional MRI. Brain Dev.

[20] Zuo XN, Xing XX (2014). Test-retest reliabilities of resting-state FMRI measurements in human brain functional connectomics: a systems neuroscience perspective. Neurosci Biobehav Rev.

[21] Zuo XN, Xu T, Milham MP (2019). Harnessing reliability for neuroscience research. Nat Hum Behav.

[22] Turner JA, Chen H, Mathalon DH, Allen EA, Mayer AR, Abbott CC (2012). Reliability of the amplitude of low-frequency fluctuations in resting state fMRI in chronic schizophrenia. Psychiatry Res.

[23] Meda SA, Wang Z, Ivleva EI, Poudyal G, Keshavan MS, Tamminga CA (2015). Frequency-specific neural signatures of spontaneous low-frequency resting state fluctuations in psychosis: evidence from bipolar-schizophrenia network on intermediate phenotypes (B-SNIP) consortium. Schizophr Bull.

[24] Bellman R (1957). Rand corporation. Dynamic programming.

[25] Hinton GE, Salakhutdinov RR (2006). Reducing the dimensionality of data with neural networks. Science.

[26] Yan CG, Wang XD, Zuo XN, Zang YF (2016). DPABI: data processing & analysis for (resting-state) brain imaging. Neuroinformatics.

[27] Euesden J, Lewis CM, O'Reilly PF (2015). PRSice: Polygenic Risk Score software. Bioinformatics.

[28] Consortium GT (2015). Human genomics. The Genotype-Tissue Expression (GTEx) pilot analysis: multitissue gene regulation in humans. Science.

[29] Xu K, Liu H, Li H, Tang Y, Womer F, Jiang X (2014). Amplitude of low-frequency fluctuations in bipolar disorder: a resting state fMRI study. J Affect Disord.

[30] Liu J, Ren L, Womer FY, Wang J, Fan G, Jiang W (2014). Alterations in amplitude of low frequency fluctuation in treatment-naive major depressive disorder measured with resting-state fMRI. Hum Brain Mapp.

[31] Pearlson GD, Clementz BA, Sweeney JA, Keshavan MS, Tamminga CA (2016). Does biology transcend the symptom-based boundaries of psychosis?. Psychiatr Clin N Am.

[32] Ivleva EI, Bidesi AS, Keshavan MS, Pearlson GD, Meda SA, Dodig D (2013). Gray matter volume as an intermediate phenotype for psychosis: Bipolar-Schizophrenia Network on Intermediate Phenotypes (B-SNIP). Am J Psychiatry.

[33] Skudlarski P, Schretlen DJ, Thaker GK, Stevens MC, Keshavan MS, Sweeney JA (2013). Diffusion tensor imaging white matter endophenotypes in patients with schizophrenia or psychotic bipolar disorder and their relatives. Am J Psychiatry.

[34] Kumar J, Iwabuchi S, Oowise S, Balain V, Palaniyappan L, Liddle PF (2015). Shared white-matter dysconnectivity in schizophrenia and bipolar disorder with psychosis. Psychol Med.

[35] Ramaker RC, Bowling KM, Lasseigne BN, Hagenauer MH, Hardigan AA, Davis NS (2017). Post-mortem molecular profiling of three psychiatric disorders. Genome Med.

[36] Huckins LM, Dobbyn A, Ruderfer DM, Hoffman G, Wang W, Pardinas AF, et al. Gene expression imputation across multiple brain regions provides insights into schizophrenia risk. Nat Genet. 2019;51:659–74. 10.1038/s41588-019-0364-4 PMC7034316 30911161

[37] Voineskos AN, Lerch JP, Felsky D, Tiwari A, Rajji TK, Miranda D (2011). The ZNF804A gene: characterization of a novel neural risk mechanism for the major psychoses. Neuropsychopharmacology.

[38] Ahmed M, Cannon DM, Scanlon C, Holleran L, Schmidt H, McFarland J (2015). Progressive brain atrophy and cortical thinning in schizophrenia after commencing clozapine treatment. Neuropsychopharmacology.

[39] Lesh TA, Tanase C, Geib BR, Niendam TA, Yoon JH, Minzenberg MJ (2015). A multimodal analysis of antipsychotic effects on brain structure and function in first-episode schizophrenia. JAMA Psychiatry.

[40] Leung M, Cheung C, Yu K, Yip B, Sham P, Li Q (2011). Gray matter in first-episode schizophrenia before and after antipsychotic drug treatment. Anatomical likelihood estimation meta-analyses with sample size weighting. Schizophr Bull.

[41] Correll CU, Rubio JM, Kane JM (2018). What is the risk-benefit ratio of long-term antipsychotic treatment in people with schizophrenia?. World Psychiatry.

[42] Wang Q, Chen R, Cheng F, Wei Q, Ji Y, Yang H (2019). A Bayesian framework that integrates multi-omics data and gene networks predicts risk genes from schizophrenia GWAS data. Nat Neurosci.

[43] Sui J, Qi S, van Erp TGM, Bustillo J, Jiang R, Lin D (2018). Multimodal neuromarkers in schizophrenia via cognition-guided MRI fusion. Nat Commun.

[44] Sui J, Jiang R, Bustillo J, Calhoun V. Neuroimaging-based individualized prediction of cognition and behavior for mental disorders and health: methods and promises. Biol Psychiatry. 2020;S0006-3223:30111–6. 10.1016/j.biopsych.2020.02.016 PMC7483317 32336400

[45] Zhi D, Calhoun VD, Lv L, Ma X, Ke Q, Fu Z (2018). Aberrant dynamic functional network connectivity and graph properties in major depressive disorder. Front Psychiatry.

