# Correction: Identifying and validating subtypes within major psychiatric disorders based on frontal–posterior functional imbalance via deep learning

**DOI:** 10.1038/s41380-020-00938-6

**Published:** 2020-11-17

**Authors:** Miao Chang, Fay Y. Womer, Xiaohong Gong, Xi Chen, Lili Tang, Ruiqi Feng, Shuai Dong, Jia Duan, Yifan Chen, Ran Zhang, Yang Wang, Sihua Ren, Yi Wang, Jujiao Kang, Zhiyang Yin, Yange Wei, Shengnan Wei, Xiaowei Jiang, Ke Xu, Bo Cao, Yanbo Zhang, Weixiong Zhang, Yanqing Tang, Xizhe Zhang, Fei Wang

**Affiliations:** 1 Department of Radiology, The First Affiliated Hospital of China Medical University, Shenyang, China; 2 Department of Psychiatry, Washington University School of Medicine, St. Louis, MO USA; 3 State Key Laboratory of Genetic Engineering and Human Phenome Institute, School of Life Sciences, Fudan University, Shanghai, China; 4 School of Computer Science and Engineering, Northeastern University, Shenyang, Liaoning China; 5 Department of Psychiatry, The First Affiliated Hospital of China Medical University, Shenyang, China; 6 Shanghai Center for Mathematical Science, Fudan University, Shanghai, China; 7 Department of Psychiatry, University of Alberta, Edmonton, AB Canada; 8 Department of Psychiatry, College of Medicine University of Saskatchewan Ellis Hall, Royal University Hospital, Saskatoon, SK Canada; 9 Department of Computer Science and Engineering, Washington University, St. Louis, MO USA; 10 Department of Genetics, Washington University School of Medicine, St. Louis, MO USA; 11 School of Biomedical Engineering and Informatics, Nanjing Medical University, Nanjing, Jiangsu China; 12 Nanjing Brain Hospital, Nanjing Medical University, Nanjing, Jiangsu China

**Keywords:** Psychology, Prognostic markers, Neuroscience

Correction to: *Molecular Psychiatry*

10.1038/s41380-020-00892-3 published online 01 October 2020

This article was originally published without open access, but has now been made available under a [CC BY 4.0] license. The PDF and HTML versions of the paper have been modified accordingly.

