# Supplementary material for: Identifying and validating subtypes within major psychiatric disorders based on frontal–posterior functional imbalance via deep learning
Source: Mol Psychiatry. 2020 Oct 1;26(7):2991–3002. doi: 10.1038/s41380-020-00892-3 (PMC8505253; doi:10.1038/s41380-020-00892-3)
Supplement: Supplementary file 1 — SupplementalMaterials [file 41380_2020_892_MOESM1_ESM.docx]

**Supplementary Information**

**Methods**

**Samples**

All participants were independently diagnosed by two trained psychiatrists using the Structured Clinical Interview for DSM-IV Axis I Disorders in participants 18 years and older and the Schedule for Affective Disorders and Schizophrenia for School-Age Children-present and Lifetime Version (K-SADS-PL) in those younger than 18 years. All patients met DSM-IV criteria for schizophrenia (SZ), bipolar disorder (BD) and major depressive disorder (MDD), and no other comorbid Axis I disorder, and healthy control (HC) participants did not have personal history of psychotic or recurrent mood disorders. Participants were excluded for (1) the presence of substance/alcohol abuse or dependence and concomitant major medical illness, (2) any magnetic resonance imaging (MRI) contraindications, and (3) history of head trauma with loss of consciousness for ≥ 5 minutes or any neurological disorders.

**MRI Acquisition**

Functional MRI, structural MRI, and diffusion tensor imaging were acquired in a GE Signa HD 3.0T scanner with a standard 8-channel head coil at the First Affiliated Hospital of China Medical University, Shenyang, China. The sequence parameters were similar to those reported previously[1, 2].

Functional images were collected with a gradient echo planar imaging (EPI) sequence. The parameters were as follows: TR = 2000ms, TE = 30ms, flip angle = 90°, field of view=240×240mm^2^, matrix = 64 × 64. Thirty-five axial slices were collected with 3mm thickness without gap. Participants were instructed to rest with their eyes closed but remain awake during scanning.

Three-dimensional, high-resolution, T1-weighted images was collected using a 3-D fast spoiled gradient-echo (FSPGR) sequence with the following parameters: TR/TE = 7.1/3.2 ms, image matrix = 240 × 240, field of view (FOV) = 240 × 240 mm^2^, 176 contiguous slices of 1 mm without gap, voxel size = 1.0 mm^3^.

DTI was acquired using a single-short spin-echo planar imaging (EPI) sequence with the following parameters: TR/TE = 17 000/85.4 ms, image matrix = 120 × 120, FOV = 240 × 240 mm^2^, 65 contiguous slices of 2 mm without gap, 25 noncollinear directions (b = 1000 s/mm^2^), together with an axial acquisition with- out diffusion weighting (b = 0), voxel size = 2.0 mm^3^.

**Functional MRI preprocessing**

Functional MRI (fMRI) preprocessing was performed with *SPM8* (http://www.fil.ion.ucl.ac.uk/spm) and *DPARSF* (http://www.restfmri.net/forum/DPARSF)[3]. For each participant, after slice-timing and realign, motion correction was assessed by means of translation/rotation. The realigned functional data were then normalized to the standard EPI template in Montreal Neurological Institute space, and resampled to 3×3×3 mm^3^. Images were spatially smoothed with a 6-mm full width at half maximum Gaussian kernel. More details on fMRI preprocessing are as reported in our previous study[2]. After preprocessing, the linear trend was removed. Then a temporal band-pass filtering (0.01-0.08 Hz) was performed to reduce the effects of low-frequency drifts and physiological high-frequency physiological noise. Amplitude of low frequency fluctuations (ALFF) at each voxel was calculated for each participant as the averaged square root of the power in the above frequency windows normalized by the corresponding mean within-brain ALFF value.

**Structural MRI and DTI preprocessing**

Structural MRI and white matter integrity preprocessing and measurement calculation were also same to our previous studies[1, 4].

Structural MRI images were processed by the Connectome Computation System (CCS: https://github.com/zuoxinian/CCS)[5], an integrated informatic platform for multimodal neuroimaging data mining and discovery sciences. The CCS processing pipeline employed in the present work included two major parts: 1) volBrain [6] implements the functions of noise removal, intensity variations correction; extraction of brain tissues; 2) FreeSurfer (version 6.0, https://surfer.nmr.mgh.harv ard.edu) implements cortical surface reconstruction with a series of functions including brain tissue segmentation, mesh tessellation and deformation to tissue boundaries, surface topological defect correction, and surface inflation into a sphere. All outcomes of the above preprocessing were visually inspected by two researchers and no participants needed manual edit. Cortical thickness and surface area were calculated in native space. Specifically, the thickness for each vertex was the mean value of twice calculation of the shortest distance between the white surface (white-gray interface) to the pial surface (gray-CSF interface) and vice versa. Cortical surface area was derived as the total area of the triangles connected to a vertex. All individual maps of cortical thickness and surface area were smoothed of 10 mm full width at half-maximum (FWHM) using a Gaussian filter, and transferred into the standard spherical surface (fsaverage).

DTI data were processed using Pipeline for Analyzing braiN Diffusion imAges (PANDA) (http://www.nitrc. org/projects/panda), a fully automated program for processing brain diffusion images. We used default program parameters to process DTI images. The voxel-wise diffusion tensor matrix was then calculated for each subject in the native space. Next, diagonalization was performed to yield 3 pairs of eigenvalues and eigenvectors. Based on the 3 eigenvalues, fractional anisotropy (FA) was computed on a voxel-by-voxel basis. Specifically, the FA image of each subject was nonlinearly registered to the FMRIB58_FA template in MNI space with 2 mm^3^ voxels. The mean of all aligned FA images was then calculated. FA images were then smoothed with a 6-mm FWHM Gaussian filter.

**Genotyping and quality control**

Genomic DNA was extracted from whole blood using the standard protocols. The samples were genotyped on the Illumina Global Screening Array-24 v1.0 BeadChip. This array provides data for 642,824 fixed genetic variants, in addition to 53,411 customized variants. For the quality control (QC), we removed single nucleotide polymorphisms (SNPs) with minor allele frequency (MAF) < 1%, call rate < 95%, or Hardy-Weinberg equilibrium p < 10^-5^. We excluded individuals with excessive missingness > 5%, gender mismatch, or an estimation of identity-by-descent > 0.9.

**Imputation and calculation of polygenic risk scores**

Genotype imputation was performed by a commercial imputation engine named GenoImpute[7]. We obtained a mean sample-level r^2^ of 0.736 estimated by 1% hold out SNPs on the array. Different to other off-the-shelf imputation engines, this engine produces a continuous allele dosage and three genotype probability distributions which reflect the reality of genotype uncertainty. Polygenetic risk scores (PRS) were calculated using PRSice software (www.PRSice.info). We used results from the latest international genomic wide association study (GWAS) published by the Psychiatric Genomics Consortium as discovery samples and our imputed genotyping data as target samples[8]. Based on the Bipolar Disorder and Schizophrenia Working Group of the Psychiatric Genomics Consortium, shared genetic factors between SZ and BD were analyzed in 53,555 SZ or BD cases combined as a single phenotype and 54,065 controls[8], and the results were used to select the SNP list for calculating PRS-SZBD. Genetic factors contributing to MDD were analyzed in 135,458 MDD cases and 344,901 controls by Wray et al.[9]; these results were used to select the SNP list for calculating PRS-MDD. We performed the p-value-informed clumping with a cutoff of r^2^=0.1 in a 250-kb window. For each set of PRS (PRS-SZBD and PRS-MDD), multiple p-value thresholds (PT) (ranging from 0 to 0.5 with increments of 0.005 plus 10^-6^, 10^-5^, 10^-4^, 10^-3^, and 1) were computed for each individual.

**Risk gene expression**

Independent GWAS was performed using PLINK1.9[10] between each subtype and HC. We then integrated genetic associations from GWAS and frontal cortex expression quantitative trait loci (eQTL) data from genotype-tissue expression (GTEx) using the web-based tool Sherlock[11, 12]. Genetic variants including cis (proximal to the gene) and trans (distal to the gene or on differing chromosome) that perturb gene expression level may influence disease risk. These expression-associated SNPs are named eSNPs. Sherlock first utilizes eQTL data from frontal cortex on whole genome-wide level to search for all eSNPs of each gene. For each searched eSNP, Sherlock will then evaluate its association with GWAS of the respective subtype (Archetypal or Atypical MPDs). Scores are evaluated in the following manner: (1) When the eSNP of a specific gene is associated with disease GWAS, a positive score is given; (2) When the eSNP of a specific gene is not associated with disease GWAS, a negative score is given; (3) When only an association seen in GWAS, the score is unchanged. The total score of a gene is the sum of scores for each eSNP. Finally, Sherlock performs gene-disease associations through utilizing a Bayes statistical framework. Bayes factor (BF, the probability of the observed data under the Bayesian statistical framework) is an indicator that evaluates evidence in favor of the assumption that the gene is associated versus not associated with the disease. For a given gene, the LBF (logarithm of BF) of each putative gene was computed and the sum of LBFs of all SNPs constitutes the total LBF score for the gene. The value of the LBF score of a gene reflects the evidence strength that this gene is associated with the disease. In other words, a larger LBF represents higher probability that this gene is associated with the disease. For more details regarding the principles for Sherlock, statistical model, and LBF calculation, please refer to the original study of He et. al.[11]. Finally, we extracted the genes with LBF > 1 (LBF > 1 provides modest evidence) from the lists of Sherlock integrative analysis where those potential risk genes were prioritized in order of LBF scores (Excel 1, Sherlock gene lists, in the Supplement). Finally, for each subtype, an expression annotation analysis for each risk gene was performed on the web-based platform FUMA (functional mapping and annotation of genetic associations) by importing the genes to the GENE2FUNC module[13], providing tissue profiles for risk gene expression in each subtype. More details on data information and analysis principles can be found in Watanabe et al.[13].

**Clinical and cognitive measures**

Hamilton Depression Rating Scale (HAMD) and Brief Psychiatric Rating Scale (BPRS) factor scores were identified from exploratory factor analysis (EFA) using the principal component factor method in major psychiatric disorders (MPDs, n=581), which results in a parsimonious list of factors using the HAMD and BPRS items. The number of factors to be extracted was determined according to the screen-plot method. Orthogonal rotation was performed using the Varimax method. Five interpretable and clinically relevant factors (Supplementary Tables 3&4) were identified which captured 62.35% of the rotated variance with a loading of 0.4.

**Building a classification model based on 3D convolutional neural network**

By using deep convolution neural network, we built a classification model (M1) based on ResNet (the 18-layer network)[14] to classify the MPDs sample (n=581 MPD) as Archetypal or Atypical MPDs. To accommodate 3D brain data, we changed all 2D convolutional kernels of the original ResNet model into 3D convolutional kernels. The network was trained from the start using random initial weight. We used cross-entropy as the loss function and Adam optimizer with ß1=0.9 and ß2= 0.999, and set the learning rate to 0.003. We trained the model for 20 epochs with a batch size of 16. Five-fold cross-validation was used for model evaluation. For each fold, we computed the accuracy, precision, recall and F1 value of the model. Confusion Matrix, the Receiver Operating Characteristic (ROC) curve and the Area Under the Curve (AUC) were also computed to evaluate the model performance. We used the same methods to build a classification model (M2) except the training labels were clinical diagnoses (SZ, BD and MDD).

**Results**

**Biological and clinical characterizations based on clinical diagnoses**

*ALFF alterations.* For the post hoc t-test, in SZ (n=193), ALFF was significantly increased in prefrontal cortex, limbic and striatum and decreased in primary sensory and motor cortices and unimodal association cortices compared to HC(n=363) (Cohen’s *d*=1.07; p<0.001) (Supplementary Figure 3). In BD (n=171), ALFF was significantly increased in prefrontal cortex and decreased in unimodal association cortices compared to HC(n=363) (Cohen’s *d*=0.54; p<0.001) (Supplementary Figure 3). In MDD (n=217), significantly decreased ALFF was found in unimodal association cortices, compared to HC(n=363) (Cohen’s *d*=0.31; p<0.001) (Supplementary Figure 3).

*Cortical thickness and white matter integrity.* In SZ and BD, cortical thickness and FA values were significantly decreased in multiple brain regions compared to HC (n=353 and 359) (cortical thickness in SZ: n=353; Cohen’s *d*=0.38; p<0.001. cortical thickness in BD: n= 359; Cohen’s *d*=0.30; p<0.001. FA values in SZ: n=186; Cohen’s *d*=0.55; p<0.001. FA values in BD: n=161; Cohen’s *d*=0.44; p<0.001) (Supplementary Figure 4). In MDD, no significant differences in cortical thickness and FA values were observed compared to HC.

*Polygenic risk scores.* All of the ten best PRS-SZBD scores [PT of 10^-4^ (N_SNPs_=1203), 0.001 (N_SNPs_=2978), 0.005(N_SNPs_=5416), 0.01 (N_SNPs_=6914, 0.015 (N_SNPs_=7802), 0.02 (N_SNPs_=8558), 0.025(N_SNPs_=9154), 0.03 (N_SNPs_=9649, 0.035(N_SNPs_=10124), 0.04 (N_SNPs_=10562)) showed significant differences between SZ and HC, explaining 6.7%, 7.1%, 8.1%, 9.9%, 10.0%, 6.4%, 5.3%, 5.7%, 5.9% and 5.5% of the variation in SZ, respectively, and remained significant after multiple comparison correction (Supplementary Figure 5). Two PRS-SZ scores at PT of 0.14 (N_SNPs_=15271), 0.15 (N_SNPs_=15633), showed significant differences between SZ and HC, explaining 5.1% of the variation in SZ, and remained significant after multiple comparison correction. No other significant differences in PRS-SZBD, PRS-BD, and PRS-MDD were found in SZ, BD and MDD.

*Risk gene expression.* SZ, BD and MDD had differential risk gene expression patterns across the 53 human tissues from GTEx[12]. For SZ, risk genes were significantly expressed in 7 tissues, one of which was brain tissue (cerebellar hemisphere) (Supplementary Figure 6a). For BD and MDD, risk genes were significantly expressed in in one non-brain tissue (heart) (Supplementary Figures 6b & c).

*Symptom severity based on medication status.* All HAMD factor scores were significantly decreased in medicated SZ (n=122) and BD (n=117), compared to their unmedicated counterparts (n=37 and n=49) (In SZ, general somatic depressive symptoms: 95% CI, 2.24-3.58; Cohen’s *d*=0.66; p=0.002. Core depressive symptoms: 95% CI, 0.57-2.94; Cohen’s *d*=0.52; p=0.004. somatization: 95% CI, 0.11-1.54; Cohen’s *d*=0.42; p=0.004. Mixed symptoms: 95% CI, 0.24-1.00; Cohen’s *d*=0.56; p=0.002. In BD, general somatic depressive symptoms: 95% CI, 0.41-2.49; Cohen’s *d*=0.46; p=0.007. Core depressive symptoms: 95% CI, 0.37-3.25; Cohen’s *d*=0.41; p=0.014. somatization: 95% CI, 0.28-2.03; Cohen’s *d*=0.43; p=0.010. Mixed symptoms: 95% CI, 0.26-0.64; Cohen’s *d*=0.24; p=0.026) (Supplementary Figure 7). Medicated SZ(n=133) had significantly decreased BPRS factors for depression/anxiety (95% CI, 0.63-3.28; Cohen’s *d*=0.47; p=0.004) and hostility/ suspicion (95% CI, 2.39-5.92; Cohen’s *d*=0.77; p<0.001) compared to unmedicated SZ (n=51) (Supplementary Figure 7). Medicated BD (n=86) had found significantly decreased BPRS factor scores for disorganized cognitive processing (n=34) (95% CI, 0.31-1.36; Cohen’s *d*=0.52; p=0.002) (Supplementary Figure 7). Medicated MDD (n=86) had significantly decreased HAMD factor scores for general somatic depressive (95% CI, 0.47-2.21; Cohen’s *d*=0.41; p=0.003) and mixed symptoms (95% CI, 0.34-1.95; Cohen’s *d*=0.39; p=0.005) compared to unmedicated MDD (n=50) (Supplementary Figure 7). There were no significant differences in BPRS factor scores between medicated and unmedicated MDD (Supplementary Figure 7).

**Building a classification model based on 3D convolutional neural network**

By using ResNet-18 convolution neural network, we built two classification models: 1) M1 to classify patients as Archetypal or Atypical MPDs and 2) M2 to classify patients as SZ, BD, or MDD. The performance of each model was evaluated based on five-fold cross-validations (Supplementary Figure 8). For M1 (subtype classification), the average accuracy was 0.89 and the precision, recall and F1 value were 0.914, 0.939 and 0.925, respectively (Supplementary Figure 8a). For M2 (clinical diagnosis classification), the average accuracy was 0.380 and the precision, recall and F1 value were 0.360, 0.371 and 0.289, respectively (Supplementary Figure 8b). The average accuracy, precision, recall and F1 value of M2 were all under 0.4, indicating that clinical diagnoses are more similar to each other and less distinguishable by the classification model. Conversely, M1 had values greater than 0.85, indicating greater distinction between the subtypes.

**Reference**

1. Chang M, Womer FY, Edmiston EK, Bai C, Zhou Q, Jiang X *et al.* Neurobiological commonalities and distinctions among three major psychiatric diagnostic categories: A structural MRI study. *Schizophr Bull* 2018; **44**(1)**:** 65-74.

2. Chang M, Edmiston EK, Womer FY, Zhou Q, Wei S, Jiang X *et al.* Spontaneous low-frequency fluctuations in the neural system for emotional perception in major psychiatric disorders: amplitude similarities and differences across frequency bands. *J Psychiatry Neurosci* 2019; **44**(2)**:** 132-141.

3. Chao-Gan Y, Yu-Feng Z. DPARSF: A MATLAB toolbox for "Pipeline" data analysis of resting-state fMRI. *Front Syst Neurosci* 2010; **4:** 13.

4. Sun Y, Wang X, Wang Y, Dong H, Lu J, Scheininger T *et al.* Anxiety correlates with cortical surface area in subjective cognitive decline: APOE epsilon4 carriers versus APOE epsilon4 non-carriers. *Alzheimers Res Ther* 2019; **11**(1)**:** 50.

5. Zuo XN, Xu T, Jiang L, Yang Z, Cao XY, He Y *et al.* Toward reliable characterization of functional homogeneity in the human brain: preprocessing, scan duration, imaging resolution and computational space. *Neuroimage* 2013; **65:** 374-386.

6. Manjon JV, Coupe P. volBrain: An Online MRI Brain Volumetry System. *Front Neuroinform* 2016; **10:** 30.

7. Wang Y, Lu J, Yu J, Gibbs RA, Yu F. An integrative variant analysis pipeline for accurate genotype/haplotype inference in population NGS data. *Genome Res* 2013; **23**(5)**:** 833-842.

8. Consortium BDaSWGotPG. Genomic dissection of bipolar disorder and schizophrenia, Including 28 subphenotypes. *Cell* 2018; **173**(7)**:** 1705-1715.

9. Wray NR, Ripke S, Mattheisen M, Trzaskowski M, Byrne EM, Abdellaoui A *et al.* Genome-wide association analyses identify 44 risk variants and refine the genetic architecture of major depression. *Nat Genet* 2018; **50**(5)**:** 668-681.

10. Purcell S, Neale B, Todd-Brown K, Thomas L, Ferreira MA, Bender D *et al.* PLINK: a tool set for whole-genome association and population-based linkage analyses. *Am J Hum Genet* 2007; **81**(3)**:** 559-575.

11. He X, Fuller CK, Song Y, Meng Q, Zhang B, Yang X *et al.* Sherlock: detecting gene-disease associations by matching patterns of expression QTL and GWAS. *Am J Hum Genet* 2013; **92**(5)**:** 667-680.

12. Consortium GT. Human genomics. The Genotype-Tissue Expression (GTEx) pilot analysis: multitissue gene regulation in humans. *Science* 2015; **348**(6235)**:** 648-660.

13. Watanabe K, Taskesen E, van Bochoven A, Posthuma D. Functional mapping and annotation of genetic associations with FUMA. *Nat Commun* 2017; **8**(1)**:** 1826.

14. Deep Residual Learning for Image Recognition. *Proceedings of the IEEE Conference on Computer Vision and Pattern Recognition*; 27-30 June 2016 2016; Las Vegas, NV, USA. IEEE2016.

**
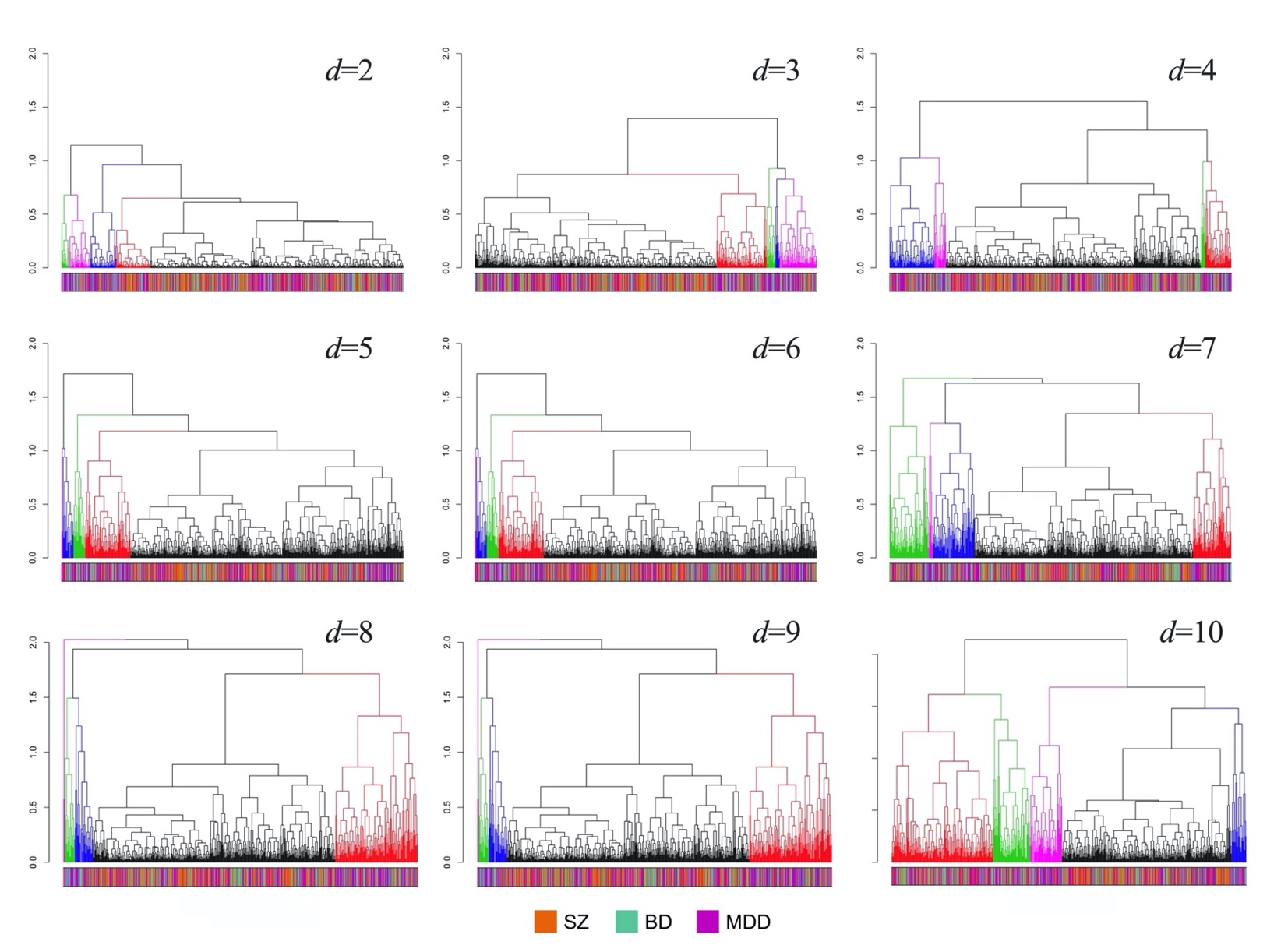
s-Figure 1.** **Hierarchical clustering analysis**

The height of each linkage in the dendrogram represents the distance between the clusters joined by that link. The color bars show the clinical diagnoses represented within the corresponding cluster. *d*, dimension. SZ, schizophrenia. BD, bipolar disorder. MDD, major depressive disorder.

**s-Figure 2. Distribution of each diagnostic category by subtypes and clinical diagnosis**

**
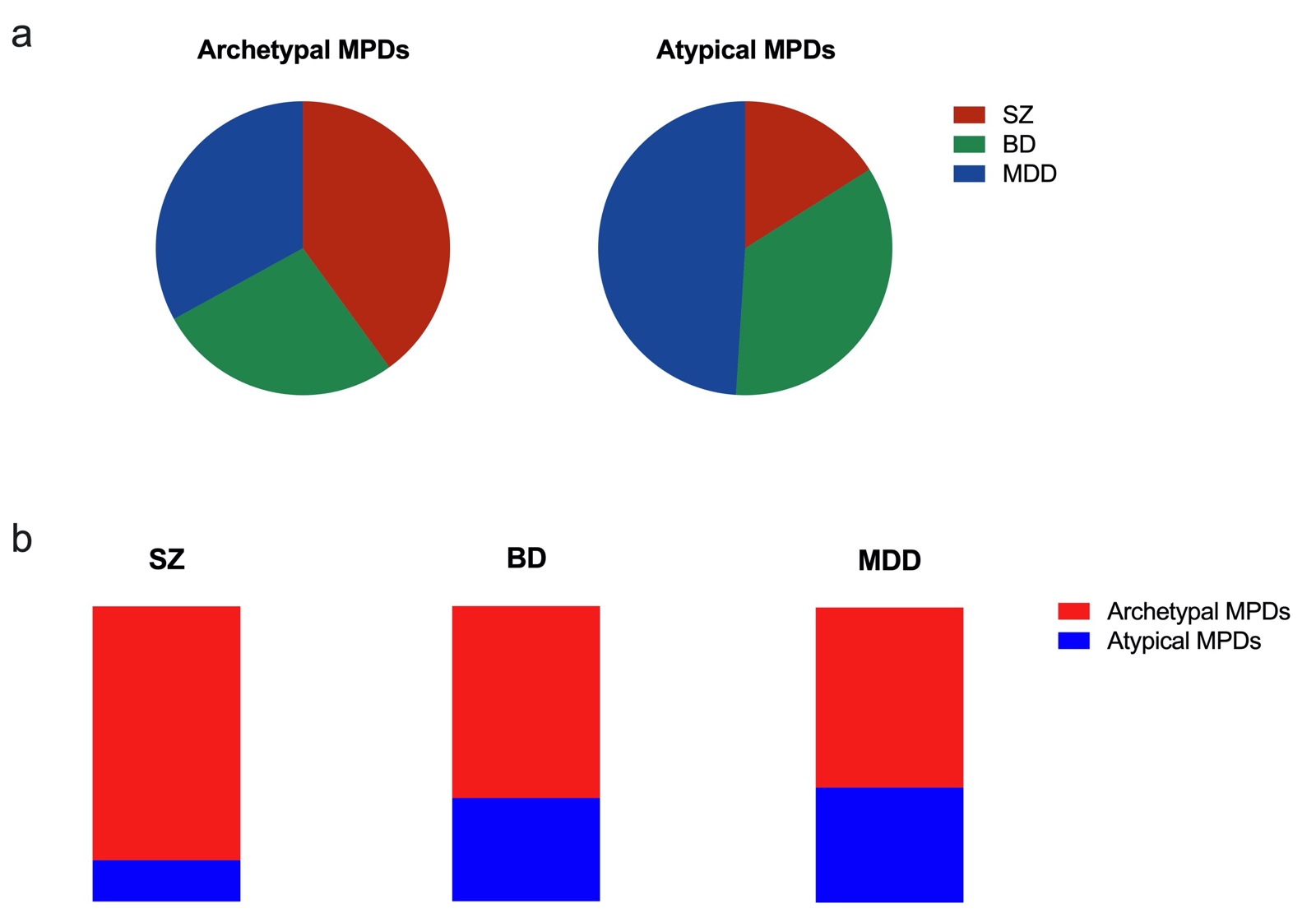
**

MPD, major psychiatric disorders. SZ, schizophrenia. BD, bipolar disorder. MDD, major depressive disorder.

**
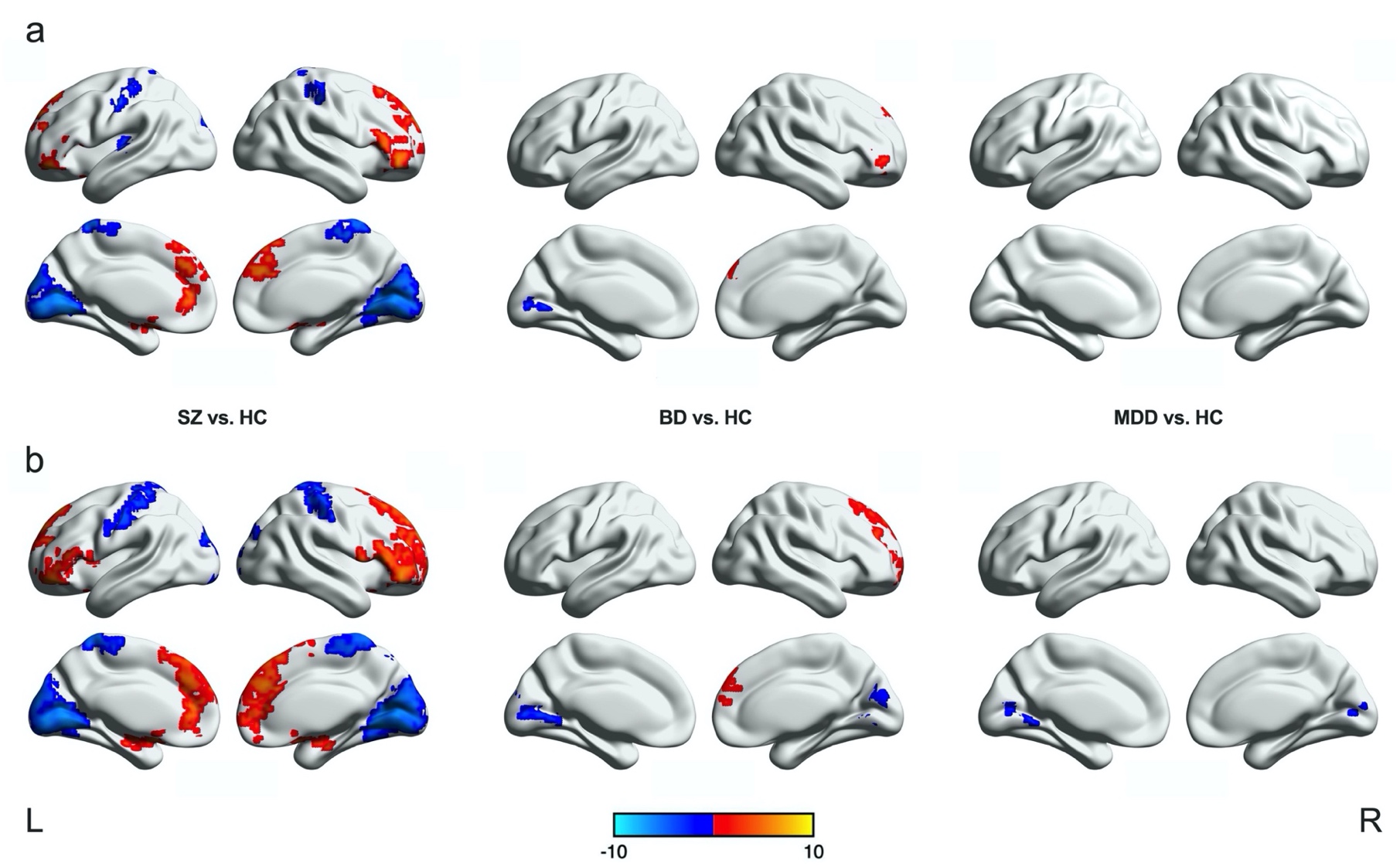
s-Figure 3.** **Significantly altered regions of** **amplitude of low-frequency fluctuation values in SZ, BD, and MDD**

(a) Significance level was set to voxel p < 0.001 with Gaussian random field (GRF) correction for cluster p < 0.05. (b) Significance level was set to voxel p < 0.01 with GRF correction for cluster p < 0.05. SZ, schizophrenia. BD, bipolar disorder. MDD, major depressive disorder. HC, healthy controls. L, left. R, right. The color bar represents t value.

**s-Figure 4. Significantly differences in (a) cortical thickness and (b) white matter integrity in SZ and BD
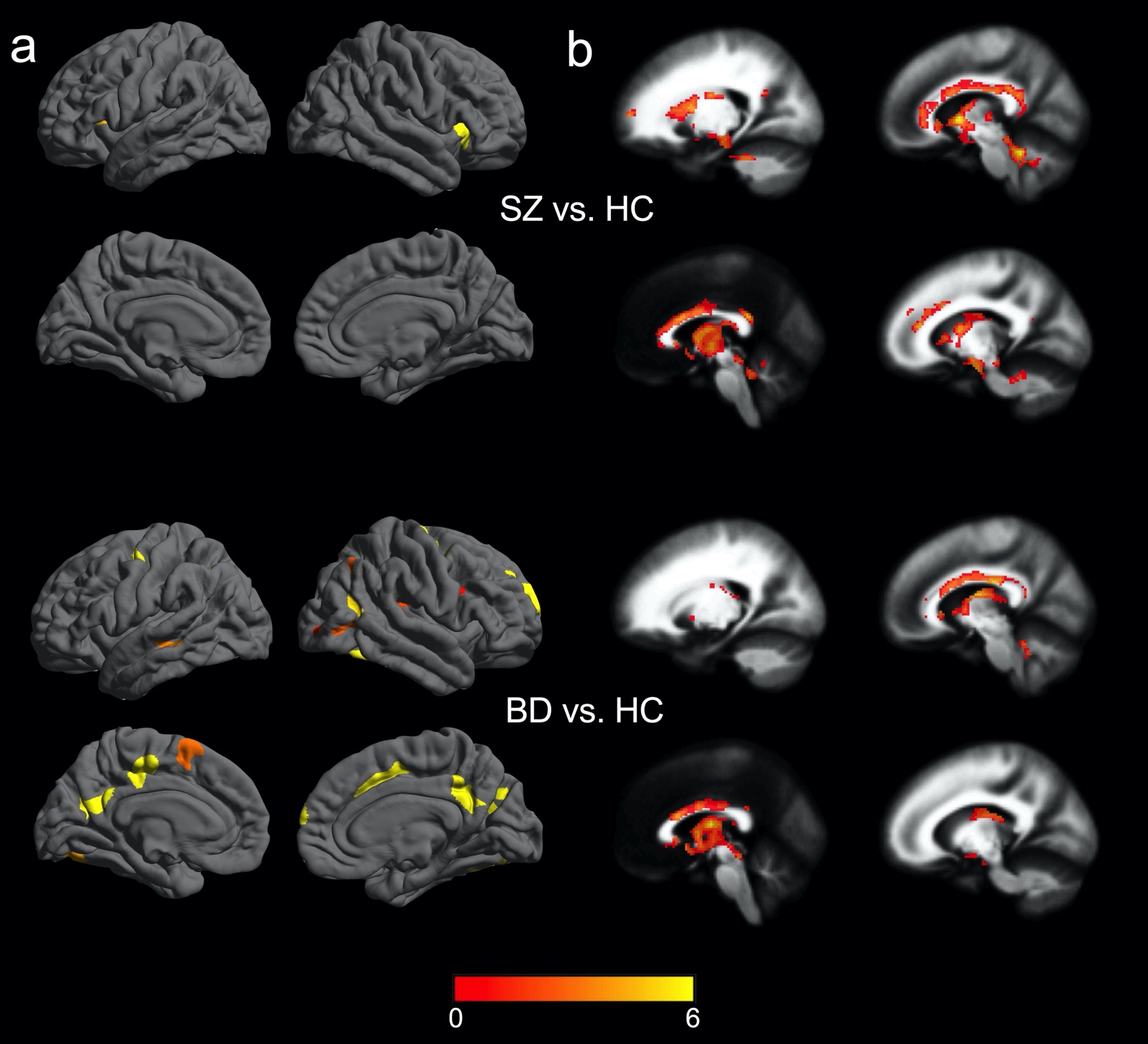
**

The significance level was set to voxel p < 0.001 with Gaussian random field correction for cluster p < 0.05. SZ, schizophrenia. BD, bipolar disorder. HCs, healthy controls. The color bar represents t value.

**
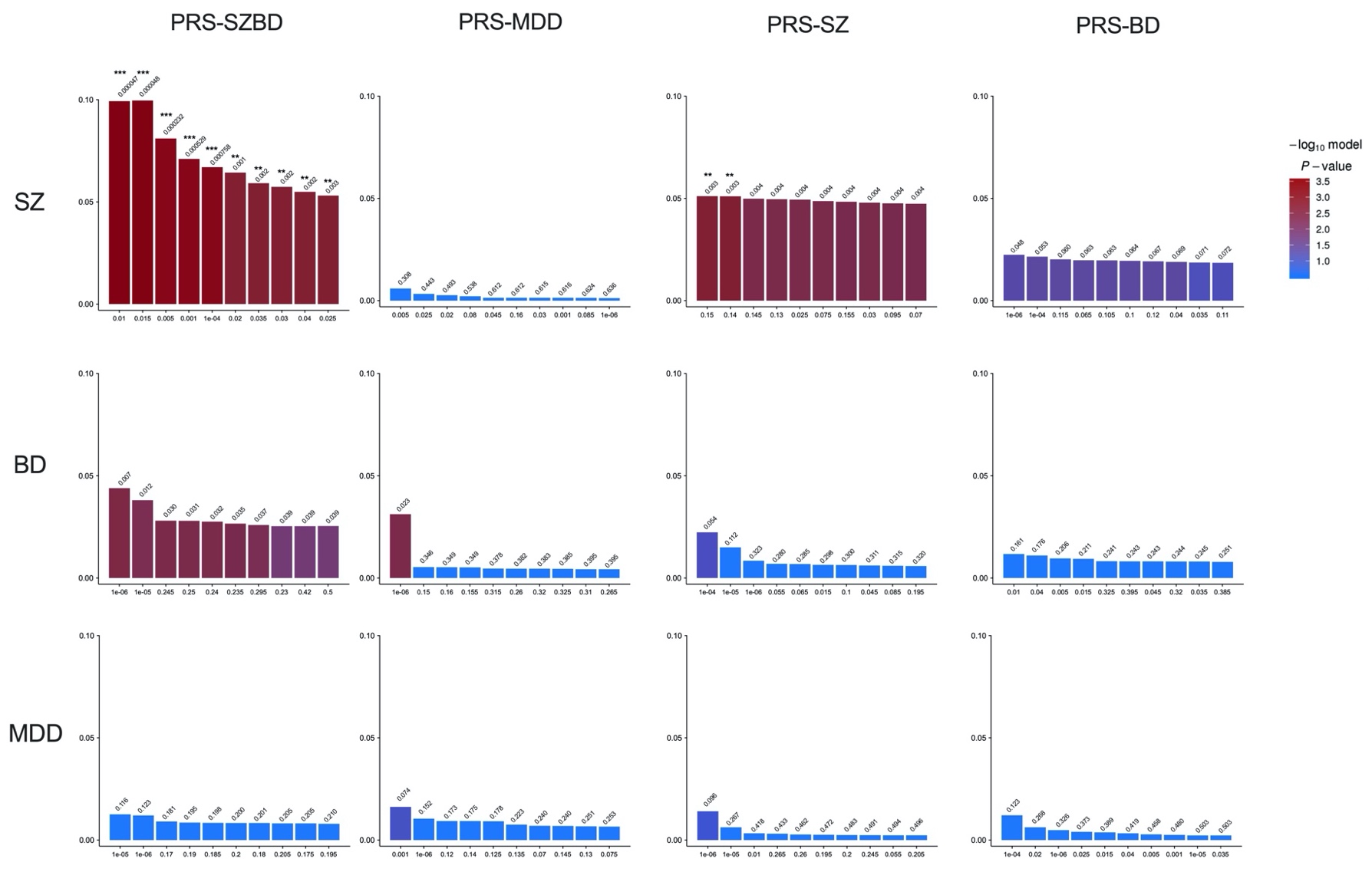
s-Figure 5. The variance (y-axis) of case-control status explained by PRS-SZ, PRS-BD, PRS-MDD and PRS-SZBD in SZ, BD and MDD**

X-axis represents P-value threshold, and y-axis represents PRS model fit: R^2^ (Nagelkerke’s). The bars represent the ten best-fit PRS scores calculated at different P-value threshold. ***, p < 0.001; **, p < 0.01. PRS-SZBD, polygenic risk score for schizophrenia and bipolar disorder, PRS-MDD, polygenic risk score for major depressive disorder.

**
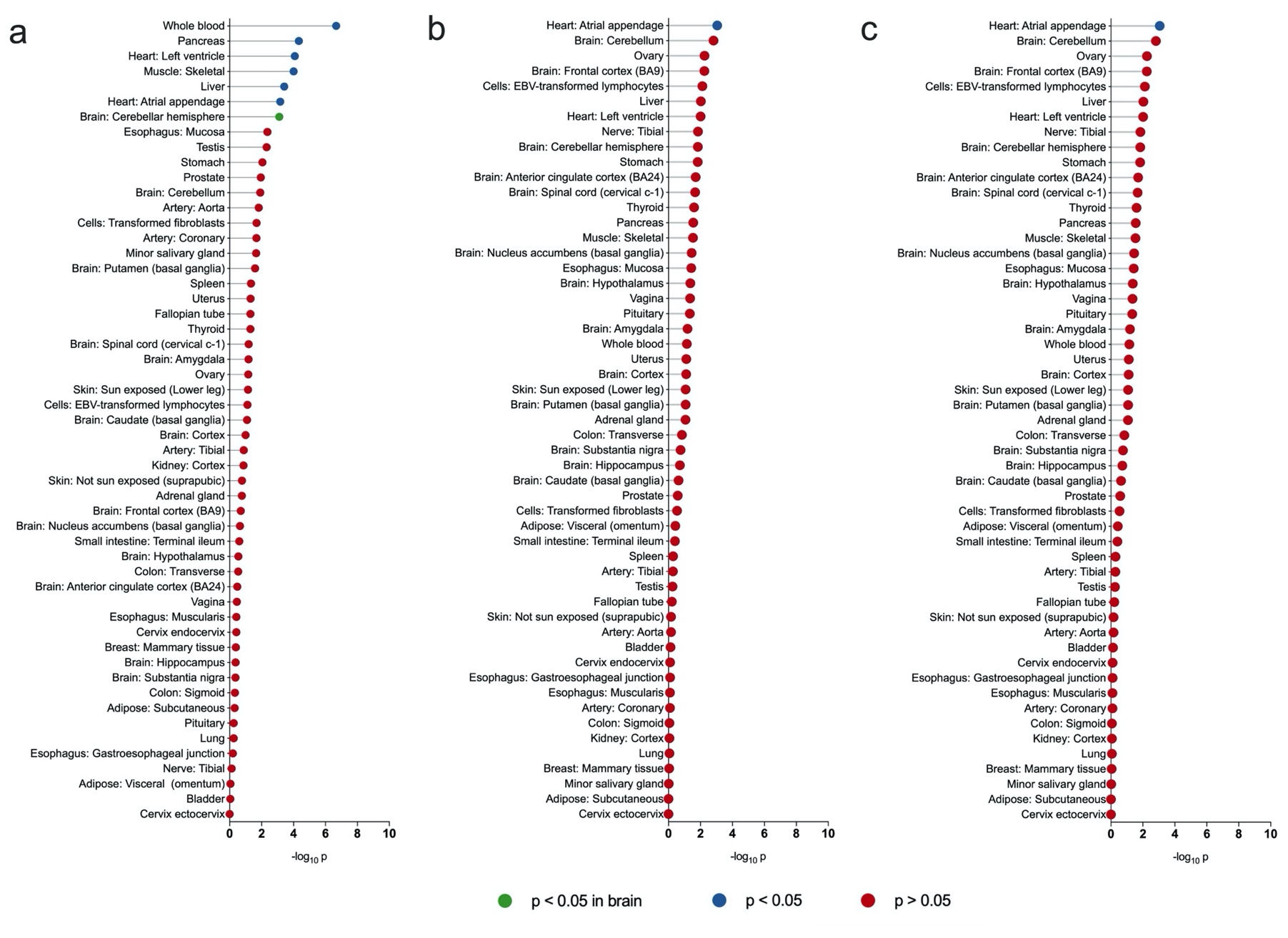
s-Figure 6. Risk gene expression profile across 53 tissues from genotype-tissue expression in (a) schizophrenia, (b) bipolar disorder, and (c) major depressive disorder**

**s-Figure 7.** **HAMD and BPRS factor scores in medicated versus unmedicated SZ, BD, and MDD**
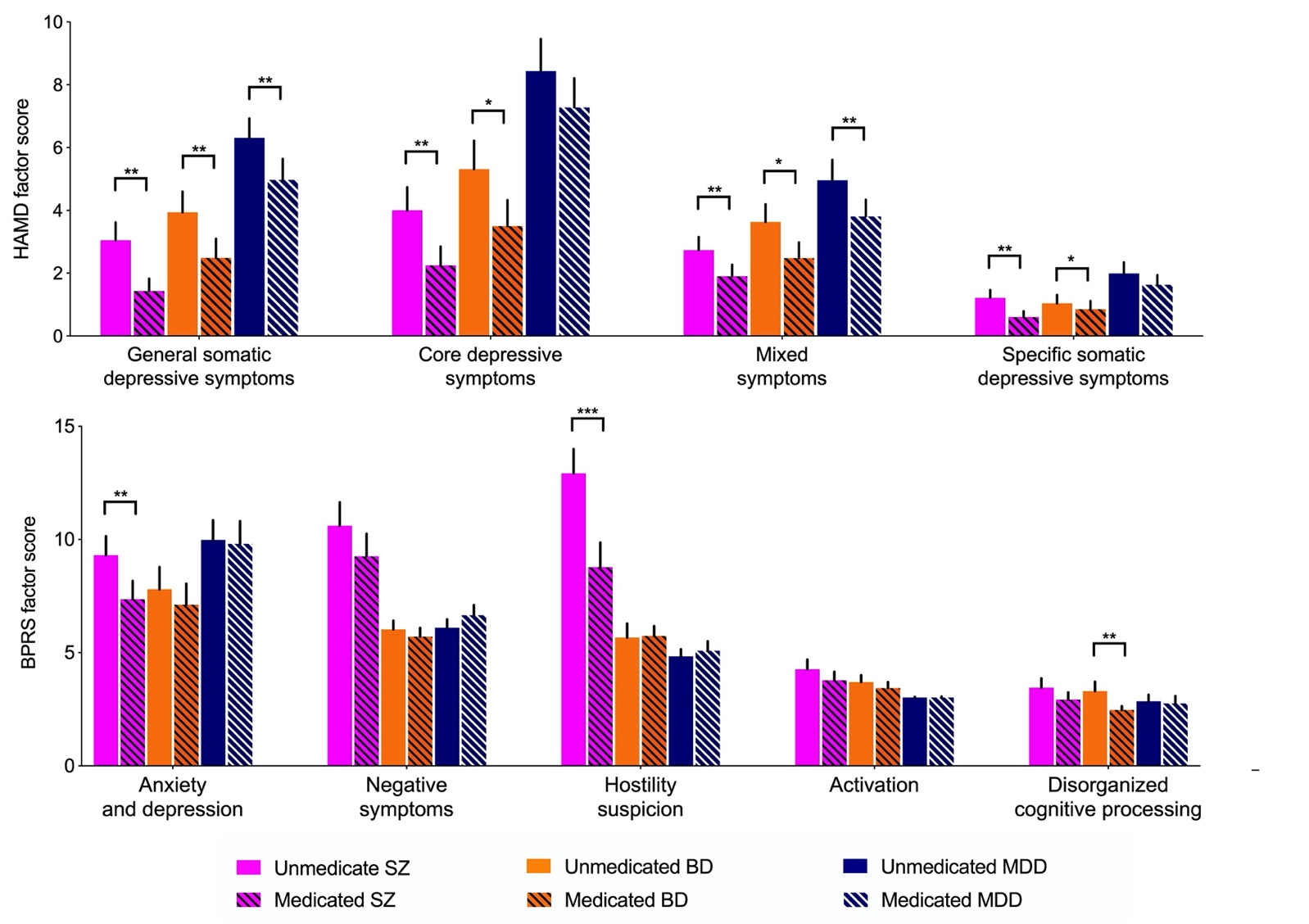


Significance level was set at p < 0.05 with false discovery rate correction. Vertical black lines show the standard errors of the means. ***, p < 0.001. **, p < 0.01. *, p < 0.05. SZ, schizophrenia. BD, bipolar disorder. MDD, major depressive disorder. HAMD, Hamilton Depression Rating Scale. BPRS, Brief Psychiatric Rating Scale.

**s-Figure 8. Performance of the classification models: (a) M1 for subtype assignment and (b) M2 for clinical diagnoses**
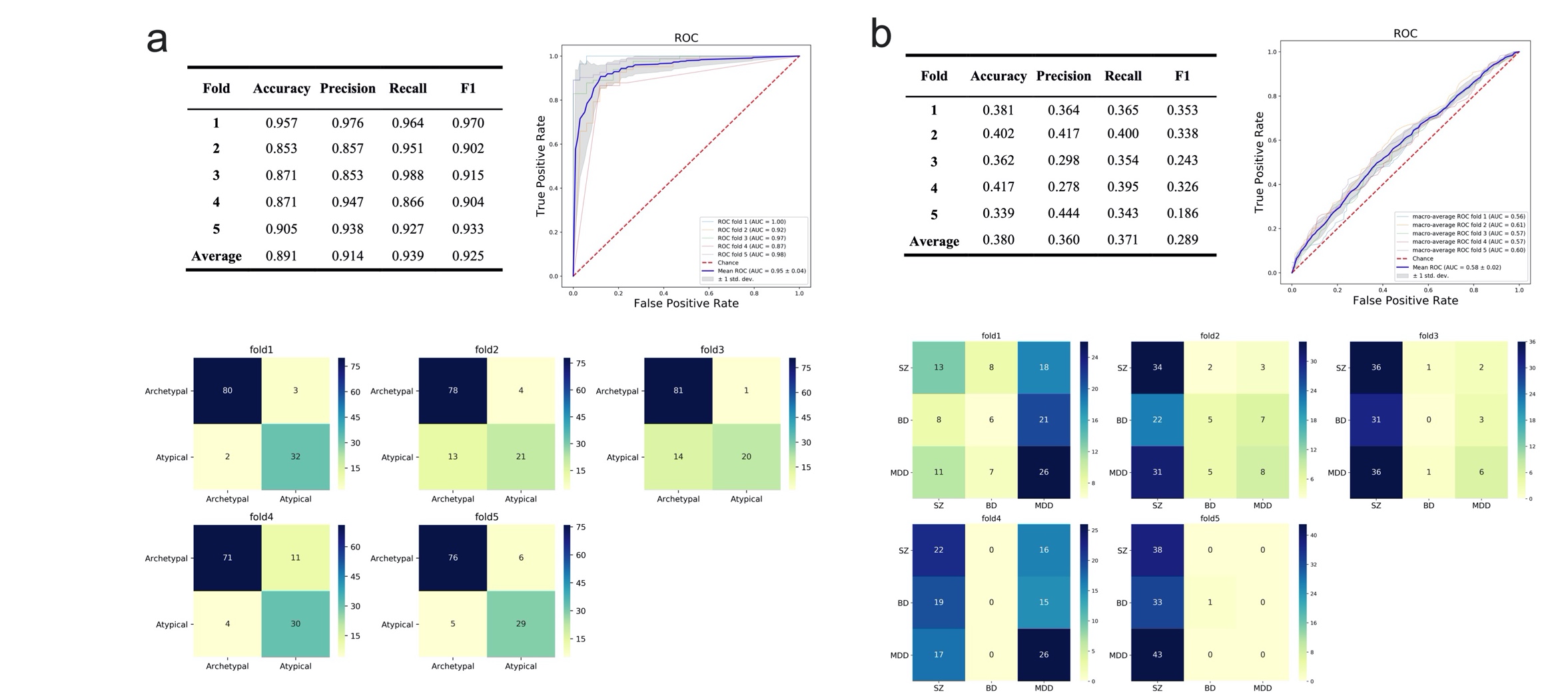


SZ, schizophrenia. BD, bipolar disorder. MDD, major depressive disorder.

|  | **Healthy controls (n=363)** | **Major psychiatric disorders (n=581)** |
| --- | --- | --- |
| Schizophrenia | - | 193 (33%) |
| Bipolar disorder | - | 171 (30%) |
| Major depressive disorder | - | 217 (37%) |
| Age at scan, years | 31.63 (12.28) | 26.31 (10.08) |
| Male | 147 (41%) | 205 (35%) |
| Duration, months | - | 30.27 (49.93) |
| First episode, yes | - | 373 (64%) |
| Medication, yes | - | 365 (63%) |
| Antipsychotics | - | 190 (33%) |
| Antidepressants | - | 180 (31%) |
| Mood stabilizer | - | 88 (15%) |
| HAMD | (n=341) | (n=540) |
|  | 1.18 (2.00) | 13.25 (10.44) |
| BPRS | (n=268) | (n=441) |
|  | 18.37 (1.10) | 29.89 (11.25) |
| WCST | (n=274) | (n=405) |
| Corrected responses | 28.09 (12.57) | 23.45 (12.06) |
| Categories completed | 3.52 (2.25) | 2.72 (2.09) |
| Total errors | 19.96 (12.65) | 24.39 (12.10) |
| Perseverative errors | 7.39 (7.21) | 10.04 (9.80) |
| Non-perseverative errors | 12.50 (7.42) | 14.4 (7.52) |

**s-Table 1. Demographic,** **clinical characteristics, and cognitive function of** **healthy controls and major psychiatric disorders**

Data are presented as either n (%) or mean (SD). Abbreviations: n, sample size. HAMD, Hamilton Depression Scale. BPRS, Brief Psychiatric Rating Scale. WCST, Wisconsin Card Sorting Test.

**s-Table 2. Demographic, clinical, and cognitive characteristics based on clinical diagnosis**

|  | **Schizophrenia**  **(n=193)** | **Bipolar disorder (n=171)** | **Major depressive disorder (n=217)** |
| --- | --- | --- | --- |
| Age at scan, years | 23.92 (9.69) | 27.26 (9.47) | 27.68 (10.53) |
| Male | 69 (36%) | 69 (40%) | 67 (31%) |
| Duration, months | 22.71 (32.14) | 49.83 (71.10) | 21.64 (37.77) |
| First episode, yes | 123 (64%) | 76 (44%) | 174 (80%) |
| Medication, yes | 137 (71%) | 119 (70%) | 109 (50%) |
| Antipsychotics | 109 (56%) | 67 (39%) | 14 (6%) |
| Antidepressants | 31 (16%) | 58 (34%) | 91 (42%) |
| Mood stabilizer | 9 (5%) | 78 (46%) | 0 |
| HAMD | (n=160) | (n=166) | (n=214) |
|  | 7.33 (6.71) | 10.67 (9.71) | 19.68 (9.81) |
| BPRS | (n=185) | (n=120) | (n=136) |
|  | 34.48 (12.05) | 25.93 (9.16) | 27.13 (7.49) |
| WCST | (n=127) | (n=119) | (n=159) |
| Corrected responses | 19.28 (11.58) | 26.33 (12.10) | 24.62 (11.58) |
| Categories completed | 1.89 (1.99) | 3.19 (2.10) | 3.03 (1.98) |
| Total errors | 28.73 (11.59) | 21.41 (12.09) | 23.16 (11.62) |
| Perseverative errors | 12.06 (10.63) | 8.39 (8.82) | 9.67 (9.60) |
| Non-perseverative errors | 16.64 (8.31) | 13.22 (7.04) | 13.48 (6.82) |

Data are presented as either n (%) or mean (SD). Abbreviations: n, sample size. HAMD, Hamilton Depression Scale. BPRS, Brief Psychiatric Rating Scale. WCST, Wisconsin Card Sorting Test.

**s-Table 3. The four-factor solution for Hamilton Depression Rating Scale**

| **General somatic depressive symptoms** | **Core depressive symptoms** | **Mixed symptoms** | **Specific somatic depressive symptoms** |
| --- | --- | --- | --- |
| Early insomnia | Depressed mood | Retardation | Gastrointestinal symptoms |
| Middle insomnia | Guilt | Agitation | Genital symptoms |
| Late insomnia | Suicide | Psychic anxiety | Weight loss |
| Somatic anxiety | Work and interests | Insight |  |
| General somatic symptoms | Hypochondria |  |  |

**s-Table 4. The five-factor solution for Brief Psychiatric Rating Scale**

| **Anxiety and depression** | **Negative symptoms** | **Hostility-suspicion** | **Activation** | **Disorganized Cognitive Processing** |
| --- | --- | --- | --- | --- |
| Anxiety | Emotional withdrawal | Hostility | Mannerisms and posturing | Somatic concern |
| Guilty | Conceptual disorganisation | Suspiciousness | Uncooperativeness | Grandiosity |
| Depression | Motor retardation | Hallucinations | Excitement |  |
| Tension | Blunted affect | Unusual thought content |  |  |
|  | Disorientation |  |  |  |

**s-Table 5. Demographic, clinical characteristics, and cognitive function of Archetypal and Atypical MPDs**

| **Variable** | **Archetypal MPDs, No. (%)**  **(n=411)** | **Atypical MPDs, No. (%)**  **(n=170)** | **P value** |
| --- | --- | --- | --- |
| Schizophrenia | 166 (40%) | 27 (16%) | - |
| Bipolar disorder | 112 (27%) | 59 (35%) | - |
| Major depressive disorder | 133 (33%) | 84 (49%) | - |
| Age, mean (SD), years | 25.87(9.98) | 27.36 (10.25) | 0.103 |
| Male | 158 (38%) | 47 (28%) | 0.013 |
| Duration, mean (SD), months | 29.90 (48.94) | 31.22 (52.57) | 0.791 |
| First episode, yes | 266 (65%) | 107 (63%) | 0.790 |
| Medication, yes | 260 (63%) | 105 (62%) | 0.758 |
| Antipsychotics | 147 (36%) | 43 (25%) | 0.014 |
| Antidepressants | 112 (27%) | 68 (40%) | 0.002 |
| Mood stabilizer | 64 (16%) | 24 (14%) | 0.656 |
| HAMD score, mean (SD) | (n=377) | (n=163) |  |
|  | 12.85 (10.49) | 14.19 (10.29) | 0.168 |
| BPRS score, mean (SD) | (n=307) | (n=134) |  |
|  | 30.20 (11.07) | 29.18 (11.67) | 0.382 |
| WCST, mean (SD) | (n=266) | (n=139) |  |
| Corrected responses | 23.35 (11.89) | 23.65 (12.42) | 0.811 |
| Categories completed | 2.70 (2.10) | 2.75 (2.10) | 0.837 |
| Total errors | 24.51 (11.95) | 24.17 (12.44) | 0.785 |
| Perseverative errors | 10.17 (9.66) | 9.81 (10.11) | 0.724 |
| Non-perseverative errors | 14.44 (7.43) | 14.30 (7.71) | 0.858 |

Data are presented as either n (%) or mean (SD). Abbreviations: n, sample size. MPD, major psychiatric disorder. HAMD, Hamilton Depression Scale. BPRS, Brief Psychiatric Rating Scale. WCST, Wisconsin Card Sorting Test.
